# Improved Plasma Lipids, Anti-Inflammatory Activity, and Microbiome Shifts in Overweight Participants: Two Clinical Studies on Oral Supplementation with Algal Sulfated Polysaccharide

**DOI:** 10.3390/md20080500

**Published:** 2022-08-02

**Authors:** Lauren A. Roach, Barbara J. Meyer, J. Helen Fitton, Pia Winberg

**Affiliations:** 1 Molecular Horizons, Illawarra Health and Medical Research Institute, School of Medical, Indigenous and Health Sciences, University of Wollongong, Wollongong, NSW 2522, Australia; 2 RDadvisor, Hobart, TAS 7006, Australia; drfitton@rdadvisor.com; 3 Venus Shell Systems Pty Ltd., Nowra, NSW 2540, Australia

**Keywords:** seaweed, metabolic syndrome, prediabetes, sulfated polysaccharide, anti-inflammation, cholesterol, Ulva, microbiome

## Abstract

Seaweed polysaccharides in the diet may influence both inflammation and the gut microbiome. Here we describe two clinical studies with an *Ulva* sp. 84-derived sulfated polysaccharide—“xylorhamnoglucuronan” (SXRG84)—on metabolic markers, inflammation, and gut flora composition. The first study was a double-blind, randomized placebo-controlled trial with placebo, and either 2 g/day or 4 g/day of SXRG84 daily for six weeks in 64 overweight or obese participants (median age 55 years, median body mass index (BMI) 29 kg/m^2^). The second study was a randomized double-blind placebo-controlled crossover trial with 64 participants (median BMI 29 kg/m^2^, average age 52) on placebo for six weeks and then 2 g/day of SXRG84 treatment for six weeks, or vice versa. In Study 1, the 2 g/day dose exhibited a significant reduction in non-HDL (high-density lipoprotein) cholesterol (−10% or −0.37 mmol/L, *p =* 0.02) and in the atherogenic index (−50%, *p =* 0.05), and two-hour insulin (−12% or −4.83 mU/L) showed trends for reduction in overweight participants. CRP (C-reactive protein) was significantly reduced (−27% or −0.78 mg/L, *p =* 0.03) with the 4 g/day dose in overweight participants. Significant gut flora shifts included increases in *Bifidobacteria*, *Akkermansia*, *Pseudobutyrivibrio*, and *Clostridium* and a decrease in *Bilophila*. In Study 2, no significant differences in lipid measures were observed, but inflammatory cytokines were improved. At twelve weeks after the SXRG84 treatment, plasma cytokine concentrations were significantly lower than at six weeks post placebo for IFN-γ (3.4 vs. 7.3 pg/mL), IL-1β (16.2 vs. 23.2 pg/mL), TNF-α (9.3 vs. 12.6 pg/mL), and IL-10 (1.6 vs. 2.1 pg/mL) (*p* < 0.05). Gut microbiota abundance and composition did not significantly differ between groups (*p* > 0.05). Together, the studies illustrate improvements in plasma lipids and an anti-inflammatory effect of dietary SXRG84 that is participant specific.

## 1. Introduction

Maintaining a healthy gut microbiome and a noninflammatory state is key to avoiding metabolic syndrome. In the studies described here, we sought to understand how the gut microbiome is affected by ingesting a type of ulvan from the green seaweed *Ulva* sp. 84, and the effects it has on plasma lipids and inflammation markers. The ulvan is referred to as SXRG84. 

The gut microbiome is a complex system of microbes necessary for digestion and homeostasis [1]. A dysregulated microbiome may lead to inflammation and gut permeability. The chronic low-grade inflammation that accompanies metabolic disorder [2] may also give rise to comorbidities such as cardiovascular disease [3], depression [4], and neuropathy [5]. Inflammatory biomarkers, such as interleukin-6 (IL-6) and high-sensitivity C-reactive protein (hsCRP) independently predict future cardiovascular events with a magnitude of effect comparable to that of low-density lipoprotein cholesterol (LDL-C). Treatments for atherosclerosis may require both inflammation inhibition and additional cholesterol reduction [6]. Gut florae respond directly and indirectly to dietary and intestinal glycans, and the microbiome is important to digestive enzyme activity, synthesis of vitamins, interaction with the immune system, interaction with pathogens, and control of inflammatory activity across the gut–blood barrier [7]. Consequently, glycans and the gut florae they support have a lifetime role on the status of the metabolic and immune system. 

Seaweeds contain large amounts of resistant dietary glycan. These include the alginates, laminarin and fucoidan (from brown seaweeds), carrageenan and agar (from red seaweeds) and “ulvans”—a diverse group of high-rhamnose-content polysaccharides from green seaweeds. This has recently been well reviewed by Shannon et al. [8]. Seaweed glycans are highly diverse [9,10]. The specificity of bacterial enzymes to dietary glucans implies that each seaweed mucopolysaccharide will have distinct effects on the composition of the microbiome and its metabolic function [11]. Consequently, a response to dietary glycans will also be specific, reflecting individual microbiome profiles, although commonalities related to the microbiome and gut processes are predicted. 

Whole *Ulva* sp. seaweeds have been shown to influence gut metabolic processes [12]. There is low toxicity in purified ulvans [13], with doses of up to 600 mg/kg of body weight over six months shown to be well tolerated in rats. *Ulva* sp. and ulvans show effective lipid-lowering qualities. More recent research has shown a consistency in these findings and a diversity in similar metabolic disease related effects from diverse algae [14,15]. 

In previous studies, brown seaweeds and their components have been shown to exert beneficial effects on allergy and inflammation [16]. The brown seaweed extract fucoidan restored gut lysozyme levels in athletes [17], but fucoidan did not affect metabolic markers in obese nondiabetic subjects [18]. Green seaweeds, including the Ulvacean species similar to the one used in this study, contain a class of polysaccharides known as “ulvans”. These heterodisperse sulfated polymers are quite diverse, but generally contain rhamnose, xylose, galactose, and uronic acid. They exhibit lipid-lowering activity [13], protective effects in irritable bowel syndrome (IBD) models [19], and antioxidant and antihyperlipidemic effects [20,21,22]. A hyperlipidemic rat model showed reductions in non-HDL-cholesterol and increases in HDL-cholesterol and a corresponding improvement in the atherogenic index (log (triglycerides/HDL-cholesterol)) [21]. At 300 mg/kg body weight, hypolipidemic effects were observed in a dose-dependent manner in high-fat-fed mice—an effect comparable to the drug simvastatin [23]. These studies were conducted primarily in animal models and human studies are warranted.

This research presents two consecutive clinical trials investigating the effects of a specific type of ulvan, sulfated xylorhamnoglucuronan, or “SXRG-84”, on the metabolic disease markers, the microbiota, and inflammation. 

The aim of Study 1 was to investigate the effects of “SXRG84” on plasma lipid levels, glucose, and insulin levels in overweight and obese individuals and assess the impact on the gut microbiota. The primary outcome measures were changes in plasma lipid levels. Secondary outcomes were carbohydrate metabolism, gut microbiota, inflammation, and oxidative stress. It was hypothesized that SXRG84 would have a favorable effect on plasma lipid levels, measures of carbohydrate metabolism, inflammation, and oxidative stress and would result in shifts in the gut microbiota when compared to the placebo group.

The second study (Study 2) was powered on the change in non-HDL-C observed in Study 1, using a randomized placebo-controlled crossover design. The primary objective was to confirm the Study 1 findings that SXRG84 would reduce non-HDL cholesterol in overweight participants. Secondary objectives were to examine the effects of the SXRG84 on further metabolic, inflammatory, and gut microbiota measures.

In this paper, we describe two studies that recruited participants who had a high BMI, and therefore, who had the potential for being metabolically challenged. The BMIs extended from 24 to 40, implying a range of metabolic flexibility. Metabolic flexibility is basically defined as being able to switch between utilizing glucose and lipids as a source of energy during fasting and fed states, as well as during exercise and resting, in order to maintain energy homeostasis [24]. A recent systematic review has shown that metabolic flexibility to glucose and insulin stimulation is inversely associated with the total amount of adipose tissue, waist circumference, and visceral adipose tissue [25]. This suggests that as the weight of a person increases, the metabolic flexibility decreases. Therefore, an additional aim of the first study is to compare metabolic markers across people that were overweight versus those that were obese on the various metabolic outcome measures as the potential to respond to treatments would be affected by metabolic flexibility.

## 2. Results

### 2.1. Participants Study 1 

Sixty-five participants in Study 1 were randomly assigned to the three blinded treatment groups. This assignment resulted in 21 participants being assigned to the placebo group, 21 participants to the 2 g dose of extract group, and 23 participants to the 4 g dose of extract group. One participant who was assigned to the 4 g dose of extract group withdrew consent due to reasons unrelated to the study (Figure S1 during the trial and so 64 participants completed the trial. Study participants had a median age of 55 years and a median BMI of 29 kg/m^2^. There were more female (*n* = 40) than male (*n* = 24) participants recruited, but the distribution of gender was not significantly different between the treatment groups (*p =* 0.26) so the population was analyzed as one. There were no significant differences at baseline between the three treatment gro ups for any of the outcome measures (Table 1).

There were no significant changes post intervention between the three treatment groups. However, given the differences in metabolic flexibility between overweight and obese participants, a secondary data analysis was conducted comparing overweight and obese participants. The baseline characteristics of overweight and obese participants only are shown in Table 2. As expected, there were significant differences between overweight and obese participants; notably an 18% increase in BMI, a 67% increase in CRP, a 47% increase in HOMA, a 36% increase in C-peptide, a 49% increase in fasting insulin, and a trend towards a 47% increase (*p =* 0.07) in insulin following a two-hour OGTT in the obese participants compared to the overweight participants. 

However, the baseline parameters between the three treatment groups (placebo, 2 g, and 4 g) did not differ in either the overweight participants or the obese participants (Table 3).

#### 2.1.1. Plasma Lipids Study 1

**Figure 1:**
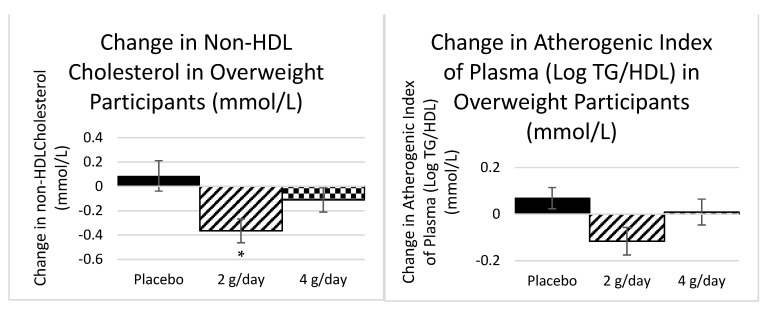
The mean change in non-HDL cholesterol and the atherogenic index of plasma after six weeks of treatment for each of the placebo and active treatments in the overweight participants. *n* = 30 (Placebo = 11, 2 g = 10, and 4 g = 9). Standard error bars shown. * Significant at *p* < 0.05.

Given the difference in metabolic flexibility between participants who are overweight and obese, a secondary analysis was conducted for the change scores calculated per treatment group for both overweight and obese groups. The overweight participants had a mean baseline total cholesterol of 5.5 mmol/L (all groups) and there was a significant decrease in non-HDL cholesterol (−10%) in the 2 g dose group (*p =* 0.02) and a trend toward a reduction in the atherogenic index (−50%) in the 2 g dose group (*p =* 0.05) (Figure 1) determined by ANOVA. There were no significant effects in the obese group who started the trial with a slightly lower baseline mean total cholesterol of 5.1 mmol/L (all groups). 

#### 2.1.2. Inflammatory Markers Study 1

**Figure 2:**
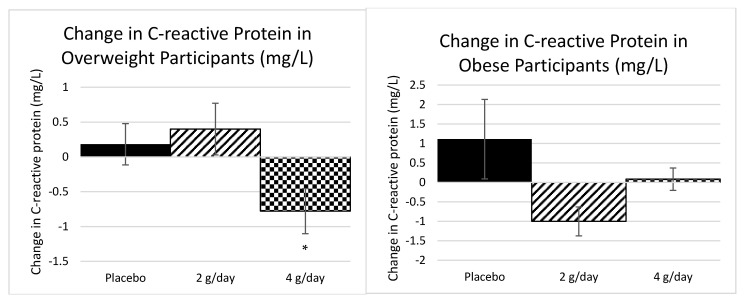
The mean change in CRP after 6 weeks of each of the three treatments for overweight *n* = 30 (Placebo = 11, 2 g = 10, and 4 g = 9) and obese participants *n* = 30 (Placebo = 9, 2 g = 9, and 4 g = 12), separately. Standard error bars shown. * Significant at *p* < 0.05.

There was a significant reduction in CRP (−27%) in the 4 g dose in the overweight participants (*p =* 0.03) and a trend towards a reduction in CRP (−27%) in the 2 g dose in the obese participants (*p =* 0.06), as determined by a Kruskal–Wallis test (Figure 2). The obese group started at a considerably higher inflammation status than the overweight group.

#### 2.1.3. Carbohydrate Metabolism Study 1

**Figure 3:**
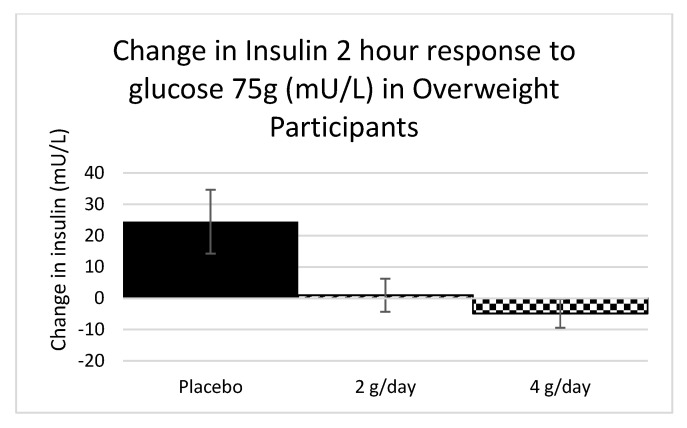
The difference in the 2-h insulin response to the OGTT for overweight participants. N = 9 for overweight 2-h insulin due to missing data (Placebo = 3, 2 g = 3, and 4 g = 3). Standard error bars shown.

There were no consistent changes in fasting glucose, fasting insulin, C-peptide, HOMA, or two-hour glucose response to the OGTT across the three treatment groups for either the overweight (*p =* 0.17, 0.34, 0.49, 0.15, and 0.86, respectively) or the obese groups (*p =* 0.86, 0.14, 0.14, 0.22, and 0.28, respectively) (ANOVA for fasting glucose and two-hour glucose; Kruskal–Wallis for remaining variables). However, there was a trend towards a reduction to the two-hour insulin response to the OGTT (−12%) in the 4 g dose for the overweight participants only (*p =* 0.05), as determined by a Kruskal–Wallis test (Figure 3).

#### 2.1.4. Microbiome Results Study 1

**Figure 4:**
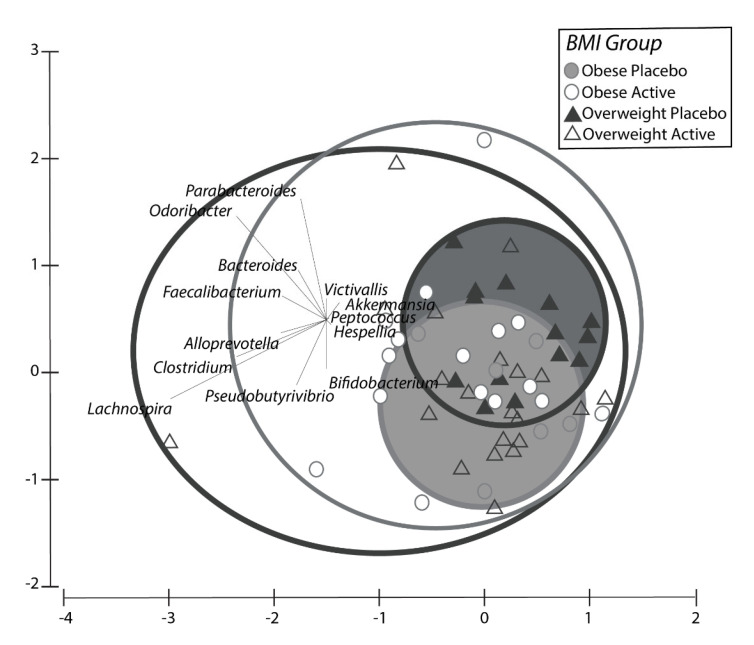
Principle component analysis of the change of composition of genera that was present in each treatment group, including Obese (light) and Overweight (dark) people on active treatments (open shapes) and placebo treatments (closed shapes). Obese Active = Obese participants on active (2 g/SXRG84 or 4 g/SXRG84), Overweight Placebo = Overweight participants on placebo, Overweight Active = Overweight participants on active (2 g/SXRG84 or 4 g/SXRG84), Obese Placebo = Obese participants on placebo.

There were significant differences (*p* < 0.05) between the pooled active treatments groups (2 g SXRG84/day and 4 g SXRG84/day combined) compared to the placebo group for both overweight and obese participants as separate groups in terms of the most-changed composition and abundance of genera of bacteria from before and after the six-week intervention. This pattern was consistent for both the overweight and the obese participant groups (Figure S3). Below, Figure 4 depicts the change in composition and abundance of genera over a six-week period between the overweight and obese participants on placebo (enclosed circles), which was much smaller compared to those on the active treatment (open circles). 

**Figure 5:**
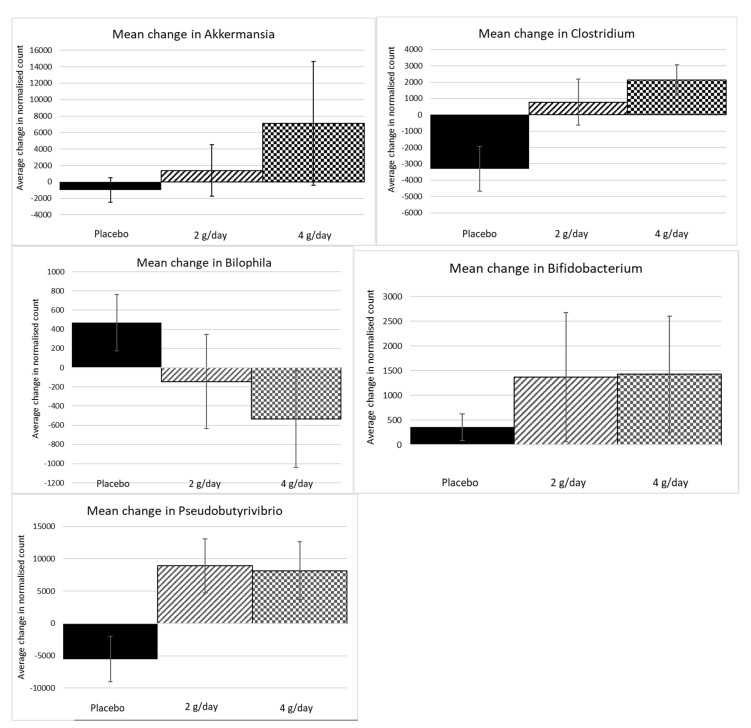
Univariate genera that were identified as those that contributed most to the difference between the active and placebo treatments consistently. Standard error bars are shown.

Permutational multivariate analysis is a powerful tool to demonstrate the overall ecological shifts of significance in the treatment and control communities of the microbiome, or the effective changes to the betadiversity. However, translating ecosystem shifts in local diversity compared to the global diversity, or betadiversity, to select species is only complicated by cascading interaction and external effects. Nevertheless, it is of interest to determine which species contributed to the significant change in betadiversity, as experiments can be designed to test cause-and-effect studies over time. Therefore, SIMPER analysis revealed 15 genera that contributed to at least 90% of the differences between placebo and pooled active group changes (Table 4). These 15 genera were examined individually to assess whether there were consistent changes in the two treatment groups compared to the placebo group. Five genera were identified that appeared to demonstrate a treatment effect. The individual genera that increased most in contrast to the placebo group were *Akkermansia*, *Clostridium*, *Pseudobutyrivibrio*, and *Bifidobacteria* (mostly *B. longum*) sp. Although not identified in the SIMPER analysis, only one genus seemed to decrease, and this was *Bilophila* sp. (Figure 5)—although the frequency of this change was not large enough to be considered one that contributed to 90% of the variation across treatments.

#### 2.1.5. Dietary Data, Bowel Movements, Urinary F_2_-Isoprostanes—Blood Count Results Study 1

Dietary intake and urinary F_2_-isoprostanes were included as a tertiary outcome in the study. Dietary intake between the three treatment groups was not significantly different at baseline for total energy (kJ), all macronutrients, sugar (g), and dietary fiber (g) and all three groups had no difference in total diet score. In the 4 g treatment group, there was a trend towards a 10% reduction in saturated fat (*p =* 0.06), as determined by ANOVA, and a trend towards a 33% reduction in added sugar as a percent of total energy after the six-week treatment, as determined by ANOVA (*p =* 0.08, Table S1, Supplementary Material).

There was no significant difference between treatment groups in regard to a change in frequency in bowel movements throughout the trial (results not shown). Safety measures such as full blood-count data (Table S4 and urinary F_2_-isoprostanes remained stable throughout the trial, with no significant changes detected between groups (*p* > 0.05) (Table S2).

### 2.2. Study 2

#### 2.2.1. Participants Study 2

Study two was a double-blind, crossover design with 70 participants randomized to either treatment regime. Each group included both active (2 g SXRG84) and placebo treatments in different orders. Six participants discontinued the intervention for the following reasons: one withdrew consent and provided no reason, one was unable to attend the final appointment due to work commitments, one was unable to attend the final appointment due to medical reasons, one did not want to continue the intervention, one fell pregnant, and one experienced a flare up of gut symptoms but was on the placebo treatment at the time. Therefore, 30 participants (15 female, 15 male) completed the placebo then treatment regime (AB) and 34 (18 female, 16 male) completed the treatment then placebo regime (BA) (Figure S2). 

At baseline there were no significant differences between the two groups (placebo then treatment (AB) or treatment then placebo (BA)). There was no significant difference in gender distribution between the two groups with 50–53% female (*p =* 0.8142) in each group (Table 5). Overall, at baseline participants had a median BMI of 29 kg/m^2^ and an average age of 52. The proportion of participants meeting the estimated average requirement (EAR) for nutrients remained constant across the three timepoints. The nutrients that were at risk (i.e., less than 50% of the study group met the EAR) were calcium, magnesium, and zinc for males and calcium for females (Table S3).

#### 2.2.2. Biochemical Analysis Study 2

There were no significant differences detected between the four groups for any of the lipid measures, blood pressure and glucose, both fasting glucose, and two-hour response to the OGTT (Table 6).

#### 2.2.3. Inflammatory Markers Study 2

Significant differences were detected between the four groups for post intervention inflammatory markers using the corresponding baseline value as a covariate (Table 7 and Table 8).

There were multiple significant changes in specific inflammatory markers, but not in CRP, bearing in mind that CRP was lower at baseline in Study 2.

#### 2.2.4. Gut Flora Results Study 2

**Figure 6:**
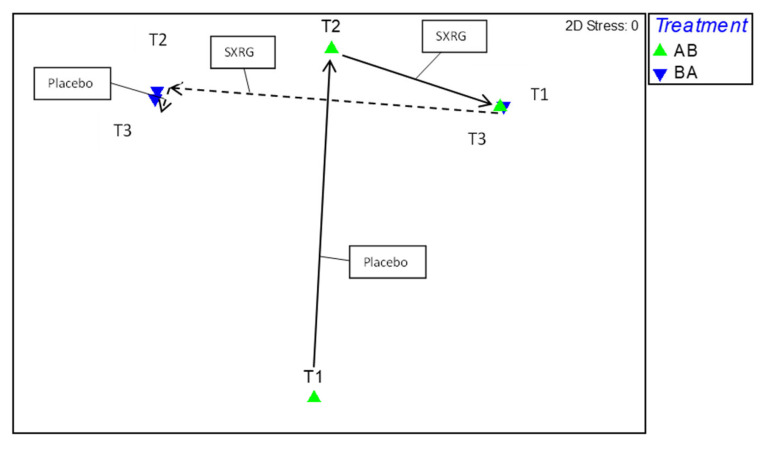
Correlation plot of gut microbiome movement per treatment group. Green triangles are regime AB (starting on placebo then crossing to SXRG84 treatment), blue triangles are regime BA (starting on SXRG84 treatment then crossing to placebo). T1 is baseline measure, T2 is measure from six weeks, and T3 is measure from 12 weeks.

A permutational multivariate analysis of variance was used to determine if there were any significant differences in gut microbiota between the two treatment regimens across the three time points, resulting in comparisons between six groups. There were no significant differences between the six groups (*p* > 0.05). A correlation plot was then used to assess the movement of gut microbiota per regime group and time point (Figure 6), on which each data point represented a time point per regime group. After visual inspection of the correlation plot, it was apparent that the two regime groups at baseline varied greatly, with movement occurring at each time point. A SIMPER analysis was conducted to determine whether there were any consistent increases or decreases in genera common to both the active treatments and not the placebo treatment. There were three genera identified that consistently changed in both regimes after the SXRG84 treatment with no effect or an opposing effect in the placebo groups—these were an increase in both *Fusicatenibacter* and *Parabacteroides* and a decrease in *Clostridium.*

There were a number of genera at baseline that significantly correlated with baseline measures of weight, BMI, waist circumference, lipids, glucose, CRP, and cytokines, which are summarized in Table 8. There were also significant correlations between the change of cytokines that were observed in this study (IFNγ, IL-1β, TNF-α, and IL-10) and the change in specific gut genera (below, Table 9). 

## 3. Discussion

This research presents two approaches to clinical studies in humans following the ingestion of a unique ulvan polysaccharide, SXRG-84. The studies explored the intervention effects on metabolic health outcomes wherein the links between lipid markers, inflammation status, and gut microbiota composition were recognized.

The primary outcome measure of Study 1 was plasma lipids, for which a significant 10% reduction in non-HDL cholesterol was observed in overweight participants. Study 2 was then powered to show the reduction in non-HDL cholesterol seen in Study 1, although this was not confirmed with potentially less metabolically challenged participants at baseline.

There was strong agreement between the two studies that dietary SXRG84 effectively reduced inflammatory markers. In the first study, the marker CRP was significantly reduced (−27%) in the 4 g/day dose group. In Study 2, a wider range of proinflammatory cytokines were reduced: IFN-γ (3.4 vs. 7.3 pg/mL), IL-1β (16.2 vs. 23.2 pg/mL), and TNF-α (9.3 vs. 12.6 pg/mL), as well as the anti-inflammatory cytokine IL-10 (1.6 vs. 2.1 pg/mL) (*p* < 0.05). These marked findings indicate a positive effect on metabolic health over a relatively short period of time. 

Each study looked at gut microbiomes. There was no consistent effect on the microbiome seen between the two studies, although Study 1 demonstrated a significant change in overall composition and abundance of microbiota in SXRG-84 treatments versus placebo, and some key biota that were important in this shift were identified. 

In more detail, Study 1 subgroups (based on BMI) presented changes in lipids, inflammation, and insulin levels and shifts in gut flora. The reduction in non-HDL cholesterol in overweight participants on the 2 g dose also showed a trend to reduction in the atherogenic index (log(triglycerides/HDL-cholesterol)). Although the changes observed in non-HDL cholesterol were much lower than those observed in statin trials [26], this group was not necessarily hypercholesterolemic to begin with. Non-HDL cholesterol is a clinically relevant target as it has strong correlations with atherogenic lipoproteins and is suggested to be a better predictor of CVD events than LDL-cholesterol [6]. 

Although Study 2 was adequately powered to show a reduction in non-HDL cholesterol, Study 1 findings were not confirmed. One reason for this null finding may be that Study 2 used a different population from Study 1. Whilst the two study populations did not differ in total cholesterol, non-HDL cholesterol, and LDL-cholesterol levels at baseline (Table 10), they differed in regard to CRP, fasting glucose, and HDL-cholesterol at baseline, with participants from Study 1 having significantly higher levels. The higher glucose and CRP indicate a slightly more metabolically challenged group in Study 1, which may have elicited a stronger treatment effect. 

The inconsistency of lipid-lowering effects highlights the need for further research with this extract, with recruitment restricted to hypercholesterolemic participants. Although Study 2 failed to show an improvement in lipid levels, the major finding was the improvements in proinflammatory cytokines after SXRG84 treatment, including IFN-γ, IL-1β, TNF-α, and the generally anti-inflammatory IL-10 [27].

Previous work from animal trials has shown that certain seaweed glycan extracts reduce plasma lipid levels through the action of bile acid sequestering [21,28,29], supported by the increase in bile acids in the feces of these animals following seaweed glycan supplementation [21]. Mechanistically, plasma lipids are lowered as they are required for the synthesis of bile acids; thus, the removal of lipids from the circulation is upregulated [21]. It was of interest that the genus Bilophila was one of the microbiotas that decreased during SXRG-84 treatment in support of this hypothesis [30,31]. Another potential reason for the lipid-lowering effects is via short-chain fatty acid propionate produced by *Bacteroides* and *Akkermansia* [32], which both increased in Study 1. Propionate production has been shown to increase up to five times more from l-rhamnose than from other sugars [33] (SXRG84 is rhamnose rich). 

In Study 1, there were significant reductions in CRP in overweight participants on 4 g of SXRG84 and a trend towards a reduction in CRP in obese participants. Although CRP is a nonspecific marker of inflammation, it is predictive of coronary heart disease and gastrointestinal diseases [34]. There was a trend towards a reduction in the two-hour insulin response to the OGTT, with no observed changes to glucose levels, suggesting an improvement in insulin sensitivity. 

In Study 2, the observed reductions in a suite of inflammatory cytokines (IFN-γ, IL-1β, and TNF-α) may be beneficial as elevated levels are implicated in metabolic and cardiovascular conditions. IFN-γ has been implicated in the development of cardiovascular disease [35]. Additionally, IL-1β and TNF-α levels are chronically raised in metabolic disease [36]. Reducing these proinflammatory cytokines may benefit overweight participants who are otherwise generally healthy but may be at increased risk. IL-10 is generally regarded as an anti-inflammatory cytokine, and it is possible that the reduction indicates a reduced inflammatory pressure [27]. In contrast, probiotic supplementation in ulcerative colitis generally decreases proinflammatory cytokines and raises IL-10 levels. 

In Study 1, the change in microbiome species composition over time (six weeks) was significantly different and more variable for participants on SXRG84 versus placebo. The impact of dietary SXRG84 on the gut flora can be summarized as an increasing shift of up to 15 taxa. In Study 1, of the 15 taxa responsible for significant differences in the active groups vs. placebo groups, 25% of the significant shift was explained by a quartet of assumed beneficial or probiotic microbiota, including *Pseudobutyrivibrio*, *Bifidobacteria*, *Akkermansia*, and *Clostridium*. These bacteria are known to respond positively to soluble dietary glycans in the distal colon. *Akkermansia* and *Bifidobacterium*, which are thought to be important for a broad range of health-related processes [37], are regarded as target organisms by researchers in the field of metabolic disease and gut health-related disorders. *Bifidobacterium* has specifically been shown to increase in response to larger molecular polysaccharides, more so than for the recognized fructo-oligosaccharides, including those with rhamnose [38]. There has been a lot of recent work focusing on the beneficial effects of probiotic *Akkermansia* intervention, and it is suggested that there is a strong synergistic relationship between the host and the bacterium in defending the gut lining and reducing leaky gut-triggered inflammation in exchange for increased mucilage production for food [39]. Achieving healthy levels of *Akkermansia* has been identified as a potential probiotic target to decrease inflammation, reduce obesity, and improve insulin sensitivity [39]. 

*Akkermansia* seems to have a high specificity, growing only on specific polysaccharides, including amine sugars, in the presence of proteins [40]. This makes sense in the case of this study, which includes amine-polysaccharides. The mechanism for protection by *Akkermansia* is still not fully understood but it is thought to relate to endocannabinoids that modulate glucose metabolism and protect against pathogenic bacteria [37,39]. *Bacteroides* was shown to decrease in all treatment groups at the largest magnitude. It is unclear as to why this occurred in all groups; however, Bacteroides is a dominant genus in the human gut, and has been shown to reflect a more western diet that is high in animal fat and protein [41]. Therefore, a reduction across the study population may suggest improved dietary habits in the participants. This, however, is only supported in the 4 g treatment group in our dietary analysis.

In Study 2, we did not observe a significant difference in gut microbiome composition between the two regimes at each timepoint. We *did* observe a consistent change in certain genera, including an increase in *Fusicatenibacter* and *Parabacteroides* and a decrease in *Clostridium* while on the SXRG84 treatment and not while on the placebo treatment for both regimes. *Parabacteroides* have increased in rats after laminarin supplementation [42], as has the species *Parabacteroides distasonis* [43], which is also a common species in rats fed with alginate. *Parabacteroides distasonis* has been identified as a laminarin fermenter [43]. It is possible, as our work has suggested, that *Parabacteroides* also responds to seaweed polysaccharides from Ulva Sp., which may infer benefits to the host. This is because *Parabacteroides* has been shown to modulate immunity [44] by suppressing the increase in inflammatory cytokines (IFN-γ, IL-12, IL-17, and IL-6) from gut tissue and increasing serum antibodies in a murine model of intestinal inflammation [44]. In Study 1, there was an increase in beneficial species of *clostridium*. In contrast, Study 2 showed a decrease in *Clostridium. Clostridium* as a genus, and species of clostridium have had different responses to seaweed extracts. In a murine model, *Clostridium cluster* XIVb and XI decreased in prevalence after laminarin supplementation [42]. Alternatively, both *Clostridium histolyticum* and *Clostridium coccoides* did not respond to ten different low-molecular-weight polysaccharides from either alginate or agar seaweeds when they were inoculated with human feces [45]. In Study 2, *Clostridium* at baseline was negatively correlated with the baseline value of the cytokines that were reduced (IFN-γ, IL-1β, TNF-α, and IL-10), suggesting an overall anti-inflammatory role of *Clostridium*. Further work is needed to determine the effects of SXRG84 extract on *Clostridium* levels across species as the current evidence is contradictory.

### Limitations and Future Work

These two studies are the first dietary clinical investigations of an ulvan, and specifically of SXRG84. They indicated a highly consistent anti-inflammatory effect. However, the lipid effects were not replicated in Study 2, possibly because of differences in the study population. 

The use of the crossover designs—as in Study 2—in randomized clinical trials are popular because they can reduce bias from confounding variables and allow participants to act as their own control. Crossover trials are appropriate for use where treatment effects are short-lived in chronic conditions [46]. However, crossover trials also have complications, including the possibility of “order effects”. In Study 2, there were no consistent effects found for either of the treatment groups or the placebo groups when examining the significant outcomes. As such, we analyzed the data as four groups instead of collapsing them into two treatment groups. The need to analyze four separate groups does undermine the strengths of running a crossover trial. The true baseline at week zero was used as a covariate in all of the metabolic and inflammatory outcome measures in an attempt to reduce any carryover effects. Study 2 did not include a washout period, with the belief that six weeks on the placebo post SXRG84 treatment would not result in any carried over benefits. Furthermore, any improvements seen after six weeks on the SXRG84 treatment should have returned to baseline after the placebo in the second arm. Indeed, the gut microbiota can revert back to their initial state within 48 h of ceasing specific diets [47]. Figure 6 suggests that the gut microbiota shift that was observed in the placebo group following the SXRG84 was minimal, and many shifts observed after six weeks on the SXRG84 reverted back in the subsequent placebo period. For example, Bacteroides decreased following the SXRG84 treatment and then increased again following the placebo. Regardless, not including a washout period can make identifying treatment effects difficult. Future crossover trials examining the gut microbiome should consider a washout period. 

Lastly, the multiple comparisons used in this study makes the likelihood of type Ⅰ errors more common and need to be considered when assessing the results. We did not adjust for multiple comparisons, as this can have the opposite effect and make type ⅠⅠ errors more common [48], therefore making it difficult to determine whether any effect exists. Due to the novel nature of this work, we would prefer to present unadjusted p-values to identify potential treatment effects.

In conclusion, Study 1 showed that the dietary inclusion of SXRG84 had a beneficial effect on a number of lipid and inflammation markers and showed a relationship broadly consistent to gut flora shifts in overweight and obese humans. The results of Study 2 failed to confirm the reduction in non-HDL cholesterol from Study 1 but did confirm the anti-inflammatory potential of SXRG84 in overweight adults across a range of specific cytokine markers. These anti-inflammatory effects may exert benefits to the host as inflammatory cytokines are interrelated with metabolic and cardiovascular diseases. Three genera (*Fusicatenibacter*, *Parabacteroides*, and *Clostridium)*, which consistently responded whilst on the SXRG84 treatment, were identified, supporting the prebiotic potential of this extract. 

Importantly, there were no changes in blood counts or other markers that indicated a compromise in health. The potential for the supplements as a preventative or additional therapy is apparent but requires further investigation. It is evident that effects will also be highly specific to an individual considering their baseline metabolic state as well as gut flora composition.

## 4. Materials and Methods

### 4.1. Study 1 Design

This double-blind randomized placebo-controlled parallel design trial was approved by the University of Wollongong Human Research Ethics Committee (approval CT13/002), and prospectively registered with the Australian New Zealand Clinical Trials Registry (ACTRN12615001057572). Sixty-four participants were recruited, with all providing written informed consent. Exclusion criteria were <18 years of age and antibiotic use in the previous two months. Participants with an overweight or obese BMI (>25 kg/m^2^) were included in the study, with one participant of a BMI of 22 kg/m^2^. Participants were randomly assigned to three treatment groups of externally identical capsules: a placebo group, a 2 g dose of seaweed extract, and a 4 g dose of seaweed extract; participants and investigators were blinded to the treatment allocations. Two treatment doses were chosen to determine if there was a dose effect, as there were no previous data on humans. Randomization to treatment groups was determined using a computer-generated sequence and groups were assigned by someone independent from the study. The trial ran for a six-week period with sampling occurring at baseline and the end of week six. Participants were advised to maintain diet and exercise routines throughout the trial period and to continue to take any medications.

### 4.2. Study 2 Design

This double-blind placebo-controlled trial was prospectively registered with the Australian New Zealand Clinical Trial Registry (ACTRN12617001010381) and approved by the University of Wollongong Human Research Ethics Committee (approval 2017/101). Participants were enrolled in the study if they were 18 years or over, had an overweight BMI (25−<30 kg/m^2^), had not recently consumed antibiotics (previous two months), and were not pregnant. 

Participants with an inflammatory skin condition were also included in the study, although this subgroup was analyzed separately (data presented in Roach et al., anticipated publication 2022).

Participants were advised to maintain their usual diet and medication regimes. The sample size was determined using a power calculation with data from Study 1 and an online calculator (https://www.stat.ubc.ca/~rollin/stats/ssize/n2.html, accessed on 7 June 2017). The change in non-HDL cholesterol that was observed in Study 1 (−0.37 mmol/L) was used as the primary outcome, the test was two-sided, with a power of 80% and alpha set at 0.05. A sample of ten per group was calculated as being statistically sufficient, with a view to recruit up to 30 per group to allow for participants that may potentially withdraw consent and missing data. Furthermore, 17 per group and 19 per group were required to detect changes in LDL-cholesterol and total cholesterol, respectively. Overweight participants were recruited as they showed the greatest response to the treatment in Study 1. A 2 g dose of SXRG84 was selected, as this dose reduced non-HDL cholesterol in Study 1.

**Figure 7:**
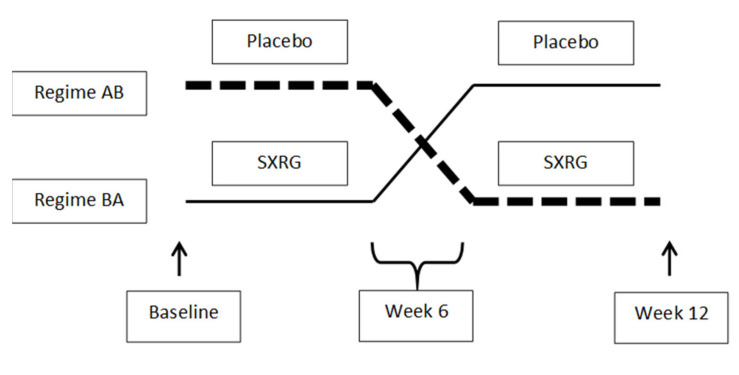
Study Design Regime AB, placebo followed by SXRG84 treatment. Regime BA, SXRG84 treatment followed by placebo.

Participants were randomized into two regimes: placebo group for six weeks and then crossed to the treatment group for six weeks (AB) or vice versa (BA) (Figure 7). Therefore, all participants consumed both the treatment and the placebo during the trial, but they were blinded as to when they consumed each treatment. Allocation of the treatment regime and labeling of the study treatments were completed by an individual independent of the study. There was no washout between the first six weeks and then second six weeks of the trial. 

### 4.3. Seaweed Extract

Sulfated xylorhamnoglucuronan-rich extract (SXRG84) from Ulva sp. 84 (PhycoDigest® Biobelly) was supplied by Venus Shell Systems Pty. Ltd. SXRG84 is an 80% pure proteoglycan extract containing 42% rhamnose, 20% glucose, and 5% xylose, as well as glucuronic and iduronic acids, and small amounts of galactose, mannose, and arabinose, with >16% sulfation and 15% protein. The molecular weight is above 600 kDa. The extract was approved for use in a clinical setting by the Australian Therapeutic Goods Administration (TGA) Clinical Trial Notification (CTN) (CT2015CTN021221) after assessment by the University of Wollongong Human Research Ethics Committee (approval CT13/002). No adverse events were recorded during the intervention. 

For Study 1, dry milled extract was formulated into 2 g and 4 g doses per 8, opaque, 0 cellulosic, vegetarian capsules, with a milled brown rice additive that was also used for the placebo capsules. Milled dark seaweed residue (without glycans) was used in trace amounts (<1%) to make all treatments visually consistent. Six-week supplies of capsules at eight per day were allocated into jars in a three-stage double-blinding system, with the key held until post-data analysis from the clinical intervention. For Study 2, the formulation was the same as described above for Study 1 and the 2 g dose was used.

Participants ingested eight capsules throughout the day and recorded daily compliance, bowel movements, and flatulence. Self-assessment compliance charts were provided at commencement and were collected at the end of the study. 

### 4.4. Blood Analysis 

Blood samples were collected after an overnight fast into EDTA tubes and placed on ice immediately for transport to laboratory facilities for spinning separation and were frozen for analysis, as outlined across the two studies below.

#### Blood Analysis Study 1

Blood samples were collected after an overnight fast into EDTA tubes by qualified phlebotomists from a professional pathology service supplier (Southern Pathology IML). Fasted blood samples were analyzed for total cholesterol, triglycerides, non-HDL cholesterol, LDL-cholesterol, CRP, glucose, C-peptide, and insulin. Samples were analyzed using a Roche Cobas 8000 or Roche Cobas Pro by Southern Pathology IML. Total cholesterol was measured using the cholesterol oxidase/peroxidase method, triglycerides were measured using the lipase/glycerol kinase method, non-HDL and LDL cholesterol were measured using the dextran sulfate/polyethylene glycol modified enzymes method, CRP was measured using a turbidimetric assay, glucose was measured using the hexokinase method, and C-peptide and insulin were measured using an electrochemiluminescence assay.

Further blood-count variables were measured for safety context and are provided in the Supplementary Material (Table S4).

#### Blood Analysis Study 2

Blood samples were collected after an overnight fast into 10 mL EDTA and 10 mL SST tubes. EDTA tubes were subjected to centrifugation within 30 min of collection for analysis of cytokines. 

EDTA plasma was analyzed by Crux Biolab (https://cruxbiolab.com.au/, accessed on 7 June 2017), using an immunoassay high sensitivity Luminex Panel for the following cytokines: IFNγ, IL-1β, IL-6, TNFα, IL-10, and IL-8. Calculated coefficients of variation (CVs) ranged from 9.6−19.3% for inter-assay variation and 0.8−7.3% for intra-assay variation. Limits for detection of the cytokines were as follows: 1.16−4770 pg/mL for IFNγ, 0.21−875 pg/mL for IL-1β, 0.74−3050 pg/mL for IL-6, 0.97−3960 pg/mL for TNFα, 0.20−810 pg/mL for IL-10, and 0.24−985 pg/mL for IL-8. 

Serum was analyzed in-house on a Konelab 20XT auto-analyzer. Commercially available kits, reagents, and standards were obtained from Thermo Fisher Scientific Australia Pty. Ltd. and were used to analyze total cholesterol (kit code 981813), HDL-cholesterol (kit code 981823), triglycerides (kit code 981786), glucose hexokinase (kit code 981779), and C-reactive protein (kit code 981934). The first four tests were colorimetric assays, while CRP was an immunoturbidimetric assay. Samples were run in singular; however, for any unusually high or low results, they were analyzed again to confirm the reading. LDL levels were calculated using the Friedwald equation (Friedewald, Levy et al., 1972) [49].

### 4.5. Oral Glucose Tolerance Test (OGTT) in Both Studies

After the fasted blood sample was collected, participants consumed a glucose drink containing 75 g of glucose. Participants then waited for 2 h and remained rested with minimal activity during this period. Then, after 2 h, another blood sample was taken into an EDTA tube via venipuncture. These samples were analyzed for glucose (Study 1 and 2) and insulin levels (Study 1). 

### 4.6. Study 1 Urine Analysis

Urine was collected by participants for a 24-h period prior to the clinic appointment in large plastic bottles provided to participants. These samples were also analyzed by the NATA-accredited pathology laboratory for creatinine, sodium, and potassium excretion. Samples were analyzed fresh in singular; any abnormal results were flagged and rerun to verify results. Samples were analyzed on a either a Roche Cobas 8000 or a Roche Cobas Pro using the creatininase method for creatinine and the ion-specific electrode-indirect method for potassium and sodium.

From this urine bottle, 1.5 mL was retained and aliquoted in cryovials. These cryovials were stored at −80 °C with no preservative prior to F_2_-isoprostane assessment using previously described methods [50]. Briefly, urine samples were thawed, acidified to a pH of 3, and internal standard was added. Separation of F_2_-isoprostanes was achieved by using silica and reverse-phase cartridges and high-performance liquid chromatography. Samples were analyzed in singular, using gas chromatography/electron capture negative-ionization mass spectrometry and the peaks were identified through the comparison of retention times with known standards. The within- and between-assay reproducibility was 6.7% and 3.7%, respectively. 

### 4.7. Gut Flora Analysis

Participants were provided with a gut testing kit by uBiome (https://en.wikipedia.org/wiki/UBiome, (131 kit numbers from November 2015 (Study 1) and 185 kit numbers June 2017 (Study 2)), which sequenced 16S rRNA of the microbiome from fecal swabs. uBiome provided the sequencing service for registered research groups which provided access to the full suite of 16s RNA raw data sets. Swabs were taken before and after the treatment interventions. Participants were required to swab a fecal sample from toilet paper using the provided swab and place the swab into a provided vial filled with a stabilization liquid. The swab was then swirled in the vial, removed, and discarded. Then, the vial was capped and stored at ambient temperature. Raw data across all taxonomic levels for each participant and time were retrieved. 

The raw taxonomic data were downloaded from Ubiome from 122 individual data sets in .json format, converted to .csv using konklone (http://konklone.io/json/, accessed on January to March 2016 for Study 1 and October 2017 to February 2018 for Study 2), and realigned in a single .xlsx database for multivariate analysis. Multivariate analysis was undertaken with PRIMER6+ using multidimensional scaling (MDS) plots to visualize patterns, which were subsequently tested statistically using PERMANOVA. Data were used as untransformed, square root-transformed, and presence/absence data to account for differences in abundance as well as composition. Data were tested using Bray Curtis similarity distance, as these are ecological data of biodiversity with a lot of zeros in the data. Where there were differences in treatment groups, SIMPER analysis was applied to determine which taxonomic group(s) contributed most to this variation. These were then tested in univariate *t*-tests using SPSS (Version 21).

The data were organized for analysis in PRIMER (version 6 with PERMANOVA) and PERMANOVA as per the following Table 11. The level of Genus provided the greatest detail of taxa without losing too much of the quantity, although overall patterns were very similar.

In the study, 6.5 million gut flora were quantified across close to 200 genera (Table 12 below). The species level of bacteria from the gut is still a field of research in rapid growth in line with the application of genetic tools, so the quantity of over 200 species is an underestimate and is the reason why only 30% of organisms can be identified at this level. Therefore, data that were analyzed at the genus level were selected as a sensitive level of taxonomy but with adequate resolution of diversity accounting for 86% of the total count, while the species level only accounted for 32% of the total count.

The data were analyzed at the taxonomic level of genus as this accounted for 86% of the total count, while species level only accounted for 32% of the total count.

Data were prepared as the change in number of normalized counts of genera for each participant. The number of genera were reduced based on the genera that contributed to the change to reduce the noise of the multivariate data or the influence of highly abundant species. Data were visually analyzed in untransformed, square root-transformed, and in presence–absence transformations in multidimensional scaling (MDS) and in principal component analysis (PCA) plots in the PRIMER-E software package (Plymouth Marine Laboratories). Permutational multivariate analysis of variance was used to compare the dissimilarities between groups using the PERMANOVA+ for PRIMER software extension. Data were analyzed in transformed and untransformed formats to determine if shifts were predominantly species abundance or composition changes. Analyses were completed using PCA and PERMANOVA models. Further identification of the genera that contributed most to the differences between groups was undertaken using the SIMPER analysis in the PRIMER-E package.

### 4.8. Dietary Intake

Study 1 participants completed a single 24-h dietary recall at the beginning and end of the six-week trial outlining all foods and beverages consumed within that 24-h period. Study 2 participants completed a single 24-h dietary recall at the beginning and again after six and 12 weeks of the trial, outlining all foods and beverages consumed within that 24-h period. These data were entered into Foodworks software (Version 7.0.3016, Xyris Software, Highgate Hill, Brisbane, Australia) for nutrient analysis.

### 4.9. Bowel Movements

The number of bowel movements were self-recorded by participants for the six-week (Study 1) and twelve-week (Study 2) trial periods on a calendar sheet. This sheet was also used to record compliance of capsule consumption. Bowel movements were counted by the investigator.

### 4.10. Statistical Analysis of Biochemical Data

The researchers were blinded for all preliminary analyses. The atherogenic index of plasma was calculated using log(triglycerides/HDL-cholesterol) (Dobiášová and Frohlich 2001) [51]. The insulin-resistance homeostasis model assessment (HOMA) index was calculated as (fasting insulin (mU/L) × fasting glucose (mmol/L))/22.5 (Matthews, Hosker et al., 1985) [52]. The OGTT was assessed by the fasting glucose measure (mmol/L) and the 2-h glucose measure (mmol/L). Participants who withdrew from the study were excluded from the statistical analysis. For those who completed the study, all participant demographics, as well as plasma, urine, and dietary data were expressed as the median (25th and 75th percentile) and were tested for normality using the Shapiro–Wilk test. Gender across the three treatment groups was compared using a chi-squared test. Baseline, finish, and change data were compared across the three treatment groups using ANOVA for normally distributed data or Kruskal–Wallis for nonparametric data. If a significant difference among the three groups existed, further post hoc tests were applied: Tukey’s test for parametric data and Dunn’s test for nonparametric data, with statistical significance assessed as *p* < 0.05. JMP Pro was used for plasma and dietary data statistical analyses.

In Study 2, SPSS Version 21 was used for analysis. For all metabolic and inflammatory outcome variables, an ANCOVA was used to test for a treatment effect of the SXRG extract on the six-week and twelve-week outcome measures using four groups. In all analyses, the baseline value was used as the only covariate to control for baseline values. The six-week to twelve-week measures were not analyzed separately due to potential carryover effects; instead, the baseline to twelve weeks was considered, which used the true baseline as a covariate.

The relationship between the different genera and the outcome variables at baseline was assessed using Spearman’s correlation. Spearman’s correlations were also used to determine whether the change in cytokines was related to the change in any specific gut microbiota genera.

## 5. Conclusions

Favorable effects on non-HDL cholesterol levels were only seen in the study population, with baseline elevated levels in Study 1. SXRG84 had a beneficial effect on inflammatory markers in overweight and obese participants. The relationship to gut flora shifts is complex, and more work is needed. Importantly, there were no changes in blood counts or other markers that indicated a compromise in health. There is potential for SXRG84 supplements to reduce inflammatory markers connected to metabolic disorders in overweight and obese participants.

## Figures and Tables

**Table 1:** Baseline demographics across treatment groups in Study 1.

	Placebo *n* = 21	2 g SXRG84/day *n* = 21	4 g SXRG84/day *n* = 22	*p*-Value
Gender, F, *n* (%)	16 (76)	11 (52)	13 (59)	0.259
Age (years)	55.0 (47.0, 60.5)	54.0 (51.0, 57.5)	54.0 (46.8, 63.3)	0.788
BMI (kg/m^2^)	29.0 (26.0, 36.0)	29.0 (27.5, 31.0)	30.0 (26.8, 33.3)	0.625
Plasma Lipids				
Total Cholesterol (mmol/L)	5.21 (4.75, 5.96)	5.05 (4.48, 5.60)	5.46 (5.06, 6.07)	0.254
Triglyceride (mmol/L) ^1^	1.07 (0.75, 1.56)	1.08 (0.76, 1.56)	1.10 (0.76, 1.82)	0.685
HDL (mmol/L) ^1^	1.52 (1.34, 2.13)	1.34 (1.20, 1.84)	1.43 (1.14, 1.93)	0.397
Cholesterol/HDL (mmol/L) ^1^	3.10 (2.55, 3.95)	3.60 (2.60, 4.10)	3.70 (2.85, 4.83)	0.265
LDL (mmol/L)	3.10 (2.35, 3.90)	3.00 (2.40, 3.45)	3.35 (2.78, 3.88)	0.412
Non-HDL (mmol/L)	3.45 (2.76, 4.35)	3.55 (3.01, 3.96)	3.84 (3.01, 4.46)	0.335
Atherogenic Index of Plasma (Log TG/HDL)	−0.16 (−0.34, 0.02)	−0.14 (−0.36, 0.12)	−0.12 (−0.32, 0.15)	0.539
Inflammation				
CRP (mg/L) *	2 (1, 4)	2 (1, 3)	3 (2, 4)	0.273
Carbohydrate Metabolism				
Fasting Glucose * (mmol/L)	5.00 (4.80, 5.15)	5.10 (4.75, 5.40)	5.15 (4.80, 5.73)	0.417
HOMA IR ^1^	2.10 (1.20, 3.09)	1.93 (1.25, 3.14)	2.11 (1.63, 4.39)	0.401
Glucose After 75 g Glucose load and 2 h ^1^ (mmol/L)	4.60 (3.55, 5.70)	5.50 (4.35, 6.50)	4.75 (4.15, 6.30)	0.110
C-Peptide ^1^ (nmol/L)	0.87 (0.53, 1.16)	0.75 (0.59, 0.98)	0.81 (0.66, 1.31)	0.492
Fasting Insulin ^1^ (mU/L)	9.30 (5.30, 14.75)	8.40 (5.60, 13.75)	9.20 (7.20, 17.78)	0.448
Insulin After 75 g Glucose load and 2 h ^1,§^ (mU/L)	29.40 (14.70, 68.50)	29.40 (20.60, 63.60)	36.30 (30.80, 129.20)	0.275

Data presented as median, 25th, and 75th percentile. SXRG84—sulfated xylorhamnogalactouronan, BMI—body mass index, HDL—high-density lipoprotein, LDL—low-density lipoprotein, TG—triglyceride, CRP—C-reactive protein, HOMA IR—Homeostatic model assessment for insulin resistance. ^1^ Log-transformed variable, * Nonparametric Kruskal–Wallis test; ^§^ *n* = 19 for 2-h insulin due to missing data (Placebo = 6, 2 g = 6 and 4 g = 7).

**Table 2:** Comparison of overweight and obese participants at baseline in Study 1.

	Overweight *n* = 30	Obese *n* = 30	*p*-Value
Sex F, *n* (%)	18 (60%)	19 (63%)	0.791
Age *	55 (49, 59)	55 (50, 63)	0.819
BMI ^1^ (kg/m^2^)	28 (26, 29)	33 (31, 38)	0.0001
Lipids			
Total Cholesterol (mmol/L)	5.5 (4.9, 6.2)	5.1 (4.5, 5.7)	0.061
Triglyceride ^1^ (mmol/L)	1.1 (0.7, 1.5)	1.2 (1.0, 1.7)	0.343
HDL ^1^ (mmol/L)	1.5 (1.3, 2.0)	1.4 (1.2, 1.7)	0.130
Chol/HDL ^1^ (mmol/L)	3.7 (2.6, 4.5)	3.6 (3.0, 4.2)	0.874
LDL (mmol/L)	3.3 (2.7, 4.1)	2.8 (2.3, 3.5)	0.115
Non-HDL (mmol/L)	3.9 (3.2, 4.7)	3.6 (3.0, 4.0)	0.210
Atherogenic Index of Plasma (Log TG/HDL)	−0.14 (−0.40, 0.11)	−0.04 (−0.26, 0.12)	0.207
CRP (mg/L) *	1 (1, 2)	3 (2, 4)	0.0001
U-Creatinine ^1^ (mmol/L)	6.3 (4.4, 8.1)	7.9 (6.0, 12.2)	0.041
U-Creatinine Excretion (mmol/d)	11.5 (8.9, 14.8)	12.2 (9.7, 18.1)	0.154
Urine Sodium Excretion (mmol/day)	104 (77, 142)	115 (87, 133)	0.586
Urine Potassium Excretion (mmol/day)	73 (60, 83)	75 (57, 88)	0.641
Na/K ^2^	1.5 (1.1, 1.9)	1.5 (1.3, 2.0)	0.749
Fasting Glucose * (mmol/L)	5.0 (4.7, 5.2)	5.3 (5.0, 5.8)	0.099
HOMA ^1^	1.7 (1.1, 2.1)	3.2 (2.0, 5.1)	0.0001
Glucose After 75 g Glucose load and 2 h ^1^	4.7 (4.0, 5.6)	5.4 (4.2, 6.5)	0.098
C-Peptide ^1^ (nmol/L)	0.7 (0.5, 0.8)	1.1 (0.9, 1.4)	0.0001
Fasting Insulin ^1^ (mU/L)	7.5 (5.1, 9.6)	14.8 (9.1, 18.1)	0.0001
Insulin After 75 g Glucose load and 2 h ^1^ (mU/L) ^§^	29.9 (24.7, 42.0)	56.5 (30.8, 129.2)	0.073

Median (25th and 75th percentile). ^1^ *t*-test on log10-transformed data. ^2^ *t*-test on square root-transformed data. * nonparametric Wilcoxon signed-rank test used for nonparametric data. ^§^ *n* = 10 overweight and 7 obese for 2-h insulin levels due to missing data.

**Table 3:** Baseline for overweight and obese participants per treatment group in Study 1.

Overweight	Placebo *n* = 11	2 g SXRG84/day *n* = 10	4 g SXRG84/day *n* = 9	*p*-Value
Baseline Total Cholesterol (mmol/L)	5.56 (4.76, 6.33)	5.15 (4.79, 5.90)	5.59 (5.28, 6.35)	0.485
Baseline LDL Cholesterol (mmol/L)	3.10 (2.40, 4.20)	3.00 (2.50, 3.65)	3.50 (3.30, 4.10)	0.347
Baseline Non-HDL Cholesterol (mmol/L)	3.41 (2.75, 4.89)	3.89 (3.19, 4.20)	4.08 (3.65, 4.82)	0.463
Baseline Triglycerides (mmol/L) ^1^	1.02 (0.62, 1.09)	1.32 (0.79, 2.10)	1.37 (0.66, 1.65)	0.288
Baseline Atherogenic Index of Plasma (Log TG/HDL)	–0.29 (–0.52, -0.10)	–0.06 (–0.37, 0.25)	–0.08 (–0.50, 0.16)	0.269
Baseline C-Reactive Protein (mg/L) *	1 (1, 2)	1 (1, 2)	2 (1, 5)	0.117
Baseline Fasting Glucose (mmol/L) *	5.00 (4.60, 5.10)	5.00 (4.68, 5.30)	4.90 (4.75, 5.15)	0.902
Baseline Glucose after 75 g Glucose Load and 2 h (mmol/L) ^1^	4.10 (3.50, 5.00)	5.50 (4.13, 6.65)	4.70 (4.20, 5.25)	0.062
Baseline Insulin 2-h Response to OGTT (mU/L) ^§^	28.30 (15.20, 30.40)	27.90 (11.50, 64.10)	36.10 (30.95, 53.48)	0.531
Baseline HOMA IR ^1^	1.30 (0.86, 2.10)	1.83 (1.21, 2.42)	1.71 (1.23, 2.11)	0.504
**Obese**	**Placebo** ***n* = 9**	**2 g SXRG84/day** ***n* = 9**	**4 g SXRG84/day** ***n* = 12**	***p*-Value**
Baseline Total Cholesterol (mmol/L) Baseline	4.92 (4.36, 5.82)	4.76 (4.01, 5.73)	5.31 (4.75, 5.92)	0.487
Baseline LDL Cholesterol (mmol/L)	2.80 (2.30, 3.50)	2.60 (2.30, 3.45)	3.00 (2.40, 3.75)	0.646
Baseline Non-HDL Cholesterol (mmol/L)	3.56 (2.85, 4.10)	3.46 (2.82, 3.93)	3.74 (3.23, 4.23)	0.442
Baseline Triglycerides (mmol/L) ^1^	1.39 (1.00, 1.71)	1.08 (0.69, 1.56)	1.10 (0.98, 1.95)	0.667
Baseline Atherogenic Index of Plasma (Log TG/HDL)	−0.01 (−0.23, 0.10)	0.00 (−0.32, 0.11)	−0.15 (−0.26, 0.22)	0.871
Baseline C-Reactive Protein (mg/L) *	4 (2, 10)	2 (2, 6)	3 (2, 4)	0.628
Baseline Fasting Glucose (mmol/L) baseline *	5.00 (4.80, 5.95)	5.30 (5.00, 5.60)	5.65 (5.15, 6.28)	0.237
Baseline Glucose After 75 g Glucose Load and 2 h (mmol/L) baseline ^1^	5.40 (4.20, 6.50)	5.50 (4.35, 6.80)	5.30 (3.83, 7.05)	0.772
Baseline Insulin 2-h Response to OGTT (mU/L) ^‡^	80.45 (56.50, 104.40)	27.20 (23.60, 30.80)	129.2 (30.8, 220.5)	0.379
Baseline HOMA IR ^1^	3.11 (2.52, 4.49)	2.73 (1.34, 3.93)	3.89 (2.16, 7.87)	0.266

Data presented as median, 25th and 75th percentile. SXRG—sulfated xylorhamnoglucuronan, HDL—high-density lipoprotein, LDL—low-density lipoprotein, TG—triglyceride, CRP—C-reactive protein, OGTT—oral glucose tolerance test. ^1^ Log-transformed variable. * Nonparametric Kruskal–Wallis test. ^§^ *n* = 9 for overweight 2-h insulin due to missing data (Placebo = 3, 2 g = 3, and 4 g = 3). ^‡^ *n* = 7 for obese 2-h insulin due to missing data (Placebo = 2, 2 g = 2, and 4 g = 3).

**Table 4:** Genera as identified in SIMPER analysis that contributed to the differences between treatment groups. * Indicates potential for treatment effect.

Genera	Contribution%	Cum.%	
Odoribacter	9.32	9.32	Increased across all groups slightly—no treatment effect
* *Akkermansia* (*muciniphila*)	9.08	18.39	Variable effect in some people.
*Lachnospira*	9.06	27.46	Significant decrease in placebo—no treatment effect
* *Clostridium*	7.95	35.40	Slight decrease in placebo—limited treatment effect
*Parabacteroides*	7.84	43.24	Variable trends—no evident treatment effect
*Faecalibacterium*	7.35	50.60	Random nonsignificant effects
* *Pseudobutyrivibrio*	6.73	57.32	Significant increases in 2 and 4 g treatments but not in placebo.
Catenibacterium	5.17	62.49	Random increase and decrease across groups
* *Bifidobacterium (longum)*	5.05	67.54	Apparent shifts in all groups but much higher in 2 g and 4 g treatments.
Desulfovibrio	5.05	72.59	Random increase and decrease across groups
Bacteroides	4.76	77.35	Decreased in all groups
Victivallis	4.28	81.64	Random increase and decrease across groups
Hespellia	4.11	85.75	Random increase and decrease across groups
Acidaminococcus	3.59	89.34	
Alloprevotella	2.29	91.63	

**Table 5:** Baseline, post (6 or 12 weeks) and change data after placebo or SXRG treatment (Study 2) for blood pressure and weight.

	AA Baseline to 6 Weeks (Placebo) *n* = 30	AB Baseline to 12 Weeks (Placebo then Active) *n* = 30	BB Baseline to 6 Weeks (Active) *n* = 34	BA Baseline to 12 Weeks (Active then Placebo) *n* = 34	*p*-Value *
Gender, F (%)	15 (50%)	15 (50%)	18 (53%)	18 (53%)	0.814
Age	51.7 ± 15	51.7 ± 15	52.2 ± 11	52.2 ± 11	0.887
BMI (kg/m^2^)					
Baseline ^§^	28 (26, 31)	28. (26, 31)	29 (27, 31)	29 (27, 31)	0.101
Post	29 (26, 31)	29 (26, 31)	29 (28, 31)	29 (27, 31)	0.363
Change	0 (0, 1)	0 (0, 0)	0 (0, 0)	0 (−1, 0)	
Waist Circumference (cm)					
Baseline	95 (85, 104)	95 (85, 104)	96 (92, 105)	96 (92, 105)	0.257
Post	97 (85, 106)	99 (87, 107)	100 (93, 106)	99 (92, 107)	0.660
Change	1 (−1, 4)	2 (−1, 5)	3 (−1, 5)	1 (−3, 4)	
Systolic BP (mmHg)					
Baseline ^§^	132 (117, 142)	132 (117, 142)	130 (122, 139)	130 (122, 139)	0.850
Post	127 (114, 141)	122 (114, 139)	129 (119, 135)	125 (114, 136)	0.809
Change	−5.0 (−10.8, 1.8)	−6.0 (−13.5, 1.0)	−4.0 (−14.5, 4.8)	−6.0 (−16.3, 0.5)	
Diastolic BP (mmHg)					
Baseline	82 (71, 89)	82 (71, 89)	83 (75, 94)	83 (75, 94)	0.524
Post	79 (72, 87)	76 (70, 85)	80 (73, 87)	79 (67, 87)	0.653
Change	−1.5 (−8.0, 2.8)	−4.0 (−8.0, 3.0)	−4.0 (−8.0, 4.0)	−3.0 (−10.8, 1.0)	

Data are presented as number and % for gender, mean ± standard deviation or median (25th and 75th percentile). AA = Placebo for 6 weeks; AB = Placebo for 6 weeks then SXRG treatment for 6 weeks; BB = SXRG treatment for 6 weeks; BA = SXRG treatment for 6 weeks then placebo for 6 weeks. Change determined by median post value (6 or 12 weeks) minus median baseline value. * *p*-value at baseline determined by *T*-test on normal or log-transformed (^§^) data between the two baseline regime groups. *p*-value for post measure determined by ANCOVA using absolute data from 6 weeks (for placebo and active group) and 12 weeks (for placebo then active group and active then placebo group) using baseline data as a covariate.

**Table 6:** Lipid and glucose baseline, post (6 or 12 weeks) and change data after placebo or SXRG84 treatment (Study 2). Data are presented as median (25th and 75th percentile). AA = Placebo for 6 weeks; AB = Placebo for 6 weeks then SXRG treatment for 6 weeks; BB = SXRG treatment for 6 weeks; BA = SXRG treatment for 6 weeks then placebo for 6 weeks.

	AA Baseline to 6 weeks (Placebo) *n* = 30	AB Baseline to 12 weeks (Placebo then Active) *n* = 30	BB Baseline to 6 weeks (Active) *n* = 34	BA Baseline to 12 weeks (Active then Placebo) *n* = 34	*p*-Value *
Total Cholesterol (mmol/L)					
Baseline	5.2 (4.5, 5.9)	5.2 (4.5, 5.9)	5.2 (4.5, 6.1)	5.2 (4.5, 6.1)	0.697
Post	4.6 (3.9, 5.4)	4.8 (4.2, 5.7)	5.3 (4.2, 5.9)	5.0 (4.4, 5.9)	0.120
Change	−0.3 (−1.0, 0.1)	−0.1 (−0.7, 0.6)	−0.1 (−0.6, 0.4)	−0.3 (−1.0, 0.2)	
HDL Cholesterol (mmol/L)					
Baseline ^§^	1.3 (1.0, 1.6)	1.3 (1.0, 1.6)	1.3 (0.9, 1.8)	1.3 (0.9, 1.8)	0.810
Post	1.1 (1.0, 1.7)	1.2 (1.0, 1.7)	1.3 (0.9, 1.9)	1.2 (0.9, 1.6)	0.493
Change	−0.0 (−0.2, 0.1)	−0.1 (−0.3, 0.2)	−0.0 (−0.2, 0.1)	−0.2 (−0.3, 0.1)	
Triglycerides (mmol/L)					
Baseline ^§^	1.0 (0.7, 1.2)	1.0 (0.7, 1.2)	1.2 (0.8, 1.8)	1.2 (0.8, 1.8)	0.112
Post	0.8 (0.7, 1.3)	1.1 (0.7, 1.3)	1.0 (0.8, 1.4)	1.0 (0.8, 1.8)	0.663
Change	−0.1 (−0.3, 0.2)	0.0 (−0.2, 0.3)	−0.1 (−0.4, 0.2)	−0.1 (−0.4, 0.1)	
Non-HDL Cholesterol (mmol/L)					
Baseline	3.7 (3.0, 4.5)	3.7 (3.0, 4.5)	4.0 (3.1, 4.4)	4.0 (3.1, 4.4)	0.651
Post	3.4 (2.6, 3.9)	3.6 (2.7, 4.3)	3.8 (3.0, 4.6)	3.7 (3.3, 4.3)	0.086
Change	−0.3 (−0.9, 0.1)	−0.1 (−0.7, 0.6)	0.0 (−0.4, 0.3)	−0.2 (−0.7, 0.4)	
LDL Cholesterol (mmol/L)					
Baseline	3.3 (2.6, 3.9)	3.3 (2.6, 3.9)	3.3 (2.6, 3.9)	3.3 (2.6, 3.9)	0.906
Post	2.9 (2.2, 3.4)	3.2 (2.2, 3.9)	3.4 (2.4, 3.7)	3.2 (2.6, 3.7)	0.103
Change	−0.3 (−0.8, 0.1)	−0.2 (−0.7, 0.5)	−0.1 (−0.5, 0.3)	−0.1 (−0.6, 0.3)	
Fasting Glucose (mmol/L)					
Baseline ^¥^	4.5 (4.1, 5.3)	4.5 (4.1, 5.3)	4.9 (4.6, 5.3)	4.9 (4.6, 5.3)	0.071
Post	4.5 (4.1, 5.0)	4.8 (4.1, 5.7)	5.0 (4.2, 5.5)	4.8 (4.3, 5.5)	0.369
Change	−0.1 (−0.4, 0.4)	0.2 (−0.4, 1.0)	−0.0 (−0.5, 0.4)	−0.1 (−0.8, 0.3)	
2-h Glucose Response to OGTT (mmol/L)					
Baseline ^¥^	4.9 (4.1, 5.7)	4.9 (4.1, 5.7)	4.8 (4.0, 6.1)	4.8 (4.0, 6.1)	0.844
Post	4.5 (4.2, 5.7)	5.1 (4.3, 5.8)	4.5 (3.8, 5.9)	5.2 (4.0, 6.8)	0.629
Change	0.0 (−0.8, 0.5)	0.0 (−0.5, 0.6)	−0.1 (−1.0, 1.0)	0.1 (−1.1, 1.2)	

Change determined by median post value (6 or 12 weeks) minus median baseline value. * *p*-value at baseline determined by *T*-test on normal, log-transformed (^§^), or Wilcoxon signed rank test (^¥^) between the two baseline regime groups. *p*-value for post measure determined by ANCOVA using absolute data from 6 weeks (for placebo and active group) and 12 weeks (for placebo then active group and active then placebo group) using baseline data as a covariate.

**Table 7:** Inflammatory markers baseline, post 6 or 12 weeks, and change data after placebo or SXRG (Study 2).

	AA Baseline to 6 Weeks (Placebo) *n* = 30	AB Baseline to 12 Weeks (Placebo then Active) *n* = 30	BB Baseline to 6 Weeks (Active) *n* = 34	BA Baseline to 12 Weeks (Active then Placebo) *n* = 34	*p*-Value *
C-Reactive Protein (mg/L)					
Baseline ^¥^	0 (0, 4.5)	0 (0, 4.5)	0 (0, 0.6)	0 (0, 0.6)	0.409
Post	0 (0, 6.9)	0.1 (0, 5.1)	0 (0, 4.5)	0 (0, 0.2)	0.240
Change	0 (0, 1.4)	0 (−0.5, 0.6)	0 (0, 3.5)	0 (0, 0)	
IFN-gamma (pg/mL)					
Baseline ^¥^	3.2 (1.8, 5.4)	3.2 (1.8, 5.4)	3.4 (1.8, 4.8)	3.4 (1.8, 4.8)	0.877
Post	3.0 (2.2, 5.8) ^a^	2.7 (1.9, 4.0) ^b^	3.6 (2.0, 4.2) ^a,b^	2.3 (1.3, 3.1) ^b^	0.014
Change	0.4 (−0.6, 1.6)	−0.4 (−2.2, 0.6)	0.2 (−1.3, 0.8)	−1.0 (−2.0, 0.0)	
IL-1 beta (pg/mL)					
Baseline ^‡^	17.5 (9.4, 27.7)	17.5 (9.4, 27.7)	14.7 (9.9, 23.3)	14.7 (9.9, 23.3)	0.547
Post	17.6 (13.0, 25.1) ^a^	15.2 (9.9, 21.7) ^b^	16.4 (13.3, 21.3) ^a,b^	10.6 (8.0, 18.3) ^b^	0.005
Change	−0.3 (−3.9, 8.9)	−1.1 (−9.0, 4.6)	1.4 (−5.9, 5.9)	−3.7 (−11.0, 0.8)	
IL-6 (pg/mL)					
Baseline ^‡^	11.8 (8.3, 20.8)	11.8 (8.3, 20.8)	12.9 (7.9, 22.3)	12.9 (7.9, 22.3)	0.896
Post	14.8 (10.5, 19.5)	12.0 (9.4, 16.5)	13.8 (9.3, 17.1)	10.3 (7.0, 16.1)	0.226
Change	1.3 ± 8.1	−2.0 ± 7.8	−1.5 ± 8.7	−2.3 ± 8.0	
TNF-alpha (pg/mL)					
Baseline ^§^	7.7 (4.0, 12.1)	7.7 (4.0, 12.1)	5.4 (2.9, 10.0)	5.4 (2.9, 10.0)	0.528
Post	8.1 (6.3, 16.9) ^a^	8.0 (3.6, 13.1) ^b^	8.7 (6.1, 11.6) ^a,b^	4.5 (2.8, 10.1) ^b^	0.005
Change	1.7 (−1.4, 7.6)	0.3 (−2.0, 3.3)	1.3 (−2.3, 5.0)	−1.2 (−6.2, 2.4)	
IL-10 (pg/mL)					
Baseline ^¥^	1.3 (0.8, 2.4)	1.3 (0.8, 2.4)	1.1 (0.7, 2.1)	1.1 (0.7, 2.1)	0.780
Post	1.5 (1.2, 2.5) ^a^	1.3 (0.8, 2.2) ^b,c^	1.6 (1.2, 2.0) ^a,b^	1.0 (0.7, 1.9) ^c^	0.009
Change	0.3 (−0.3, 1.1)	0.0 (−0.3, 0.5)	0.1 (−0.4, 0.8)	−0.2 (−0.9, 0.3)	
IL-8 (pg/mL)					
Baseline ^§^	5.2 (3.5, 10.1)	5.2 (3.5, 10.1)	4.7 (2.7, 8.5)	4.7 (2.7, 8.5)	0.607
Post	5.7 (3.9, 8.8)	3.9 (3.0, 6.9)	5.2 (4.0, 7.0)	3.9 (1.7, 9.2)	0.254
Change	0.2 (−1.3, 3.0)	−1.1 (−2.8, 0.2)	−0.2 (−2.6, 2.6)	−0.5 (−2.1, 0.9)	

Data are presented as median (25th and 75th percentile). AA = Placebo for 6 weeks; AB = Placebo for 6 weeks then SXRG treatment for 6 weeks; BB = SXRG treatment for 6 weeks; BA = SXRG treatment for 6 weeks then placebo for 6 weeks. Change determined by median post value (6 or 12 weeks) minus median baseline value. * *p*-value at baseline determined by T-test on normal, log-transformed (^§^), square root-transformed data (^‡^), or Wilcoxon signed rank test (^¥^) between the two baseline regime groups. *p*-value for post measure determined by ANCOVA using absolute data from 6 weeks (for placebo and active group) and 12 weeks (for placebo then active group and active then placebo group) using baseline data as a covariate. Values with different superscript letters denotes statistical significance (*p* < 0.05).

**Table 8:** Significant correlations with baseline measures and gut microbiota genera in Study 2.

Baseline Variable	Genus	Spearman’s Correlation Coefficient	*p*-Value
Weight (kg)	*Flavonifractor*	−0.282	0.039
	*Intestinibacter*	−0.413	0.002
	*Megasphaera*	0.587	0.001
	*Thalassospira*	−0.538	0.001
BMI (kg/m^2^)	*Anaerotruncus*	−0.295	0.027
	*Blautia*	−0.289	0.028
	*Intestinibacter*	−0.325	0.016
	*Megasphaera*	−0.519	0.004
	*Ordoribacter*	0.290	0.048
	*Romboutsia*	0.288	0.047
Waist Circumference (cm)	*Anaerotruncus*	−0.360	0.007
	*Bifidobacterium*	−0.315	0.048
	*Flavobacterium*	−0.333	0.044
	*Flavonifractor*	−0.363	0.007
	*Intestinibacter*	−0.479	0.000
	*Megasphaera*	0.476	0.009
	*Thalassospira*	−0.464	0.005
CRP	*Erysipelatoclostridium*	0.297	0.031
	*Parasutterella*	0.398	0.010
	*Subdoligranulum*	0.317	0.017
Total Cholesterol	*Megasphaera*	−0.375	0.045
	*Mogibacterium*	−0.313	0.032
	*Parabacteroides*	−0.303	0.025
HDL Cholesterol	*Anaerotruncus*	0.318	0.017
	*Intestinibacter*	0.308	0.024
	*Megasphaera*	−0.482	0.008
	*Thalassospira*	0.519	0.001
Triglycerides	*Anaerotruncus*	−0.328	0.014
	*Barnesiella*	−0.365	0.015
	*Corynebacterium*	0.373	0.016
	*Flavobacterium*	−0.362	0.025
	*Flavonifractor*	−0.315	0.020
	*Intestinibacter*	−0.433	0.001
	*Pseudoflavonifractor*	−0.422	0.010
	*Sarcina*	−0.360	0.006
	*Thalassospira*	−0.380	0.022
Non-HDL Cholesterol	*Parabacteroides*	−0.330	0.014
LDL Cholesterol	*Collinsella*	−0.272	0.045
	*Parabacteroides*	−0.325	0.015
Fasting Glucose	*Alloprevotella*	−0.529	0.043
	*Bacteroides*	0.278	0.034
	*Erysipelatoclostridium*	0.289	0.036
	*Flavonifractor*	−0.480	0.000
	*Haemophilus*	−0.362	0.010
	*Intestinibacter*	−0.277	0.043
	*Megamonas*	−0.810	0.015
	*Pseudoflavonifractor*	−0.504	0.002
	*Subdoligranulum*	0.280	0.036
	*Veillonella*	−0.305	0.030
2-h Glucose Post Oral Glucose Tolerance Test	*Haemophilus*	−0.374	0.014
	*Intestinibacter*	−0.284	0.048
IFNγ	*Clostridium*	−0.271	0.045
	*Granulicatella*	0.467	0.012
IL-1β	*Actinomyces*	0.301	0.034
	*Clostridium*	−0.351	0.008
	*Corynebacterium*	0.324	0.039
	*Granulicatella*	0.447	0.015
	*Prevotella*	0.330	0.028
IL-6	*Adlercreutzia*	0.300	0.048
	*Corynebacterium*	0.348	0.028
	*Granulicatella*	0.386	0.042
TNF-α	*Clostridium*	−0.321	0.017
	*Granulicatella*	0.428	0.023
	*Prevotella*	0.354	0.018
IL-10	*Clostridium*	−0.321	0.016
	*Corynebacterium*	0.370	0.017
	*Granulicatella*	0.428	0.020
	*Parabacteroides*	−0.280	0.038
	*Prevotella*	0.361	0.016
	*Thalassospira*	−0.446	0.006
IL-8	*Adlercreutzia*	0.311	0.042
	*Alistipes*	0.296	0.035
	*Bifidobacterium*	0.317	0.049
	*Flavonifractor*	0.278	0.046
	*Granulicatella*	0.464	0.015

**Table 9:** Cytokines that significantly changed in Study 2 correlate with a change in specific gut microbiota genera.

Cytokine	Genus	Spearman Correlation Coefficient	*p*-Value
IFN-γ	*Collinsella*	−0.298	0.023
	*Oscillibacter*	−0.297	0.023
	*Romboutsia*	−0.278	0.034
IL-1β	*Collinsella*	−0.299	0.023
	*Lactobacillus*	−0.289	0.028
TNF-α	*Bacteroides*	−0.332	0.011
	*Dorea*	−0.320	0.014
	*Peptococcus*	0.270	0.040
IL-10	*Bacteroides*	−0.369	0.004
	*Dorea*	−0.300	0.022

**Table 10:** Comparison of baseline data from Study 1 to Study 2.

	Study 1 *n* = 64	Study 2 *n* = 64	*p*-Value
Gender, F, M (%)	42, (63%)	33, (52%)	0.198
Age (years)	54 ± 10	52 ± 13	0.326
Weight (kg) ^§^	85 (74, 101)	84 (76, 95)	0.584
BMI ^¥^ (kg/m^2^)	29 (26, 33)	29 (27, 31)	0.471
Total Cholesterol (mmol/L)	5.3 ± 0.9	5.3 ± 1.2	0.750
HDL Cholesterol (mmol/L) ^‡^	1.4 (1.2, 2.0)	1.3 (1.0, 1.7)	0.007
Non-HDL Cholesterol (mmol/L) ^‡^	3.6 (3.1, 4.1)	3.9 (3.0, 4.4)	0.180
LDL Cholesterol (mmol/L) ^‡^	3.0 (2.4, 3.6)	3.3 (2.6, 3.9)	0.143
Triglycerides (mmol/L) ^§^	1.1 (0.7, 1.6)	1.1 (0.8, 1.6)	0.898
Fasting Glucose (mmol/L) ^¥^	5.1 (4.8, 5.4)	4.8 (4.3, 5.2)	0.004
Glucose 2-h Post OGTT (mmol/L) ^¥^	4.7 (4.0, 6.3)	4.8 (4.0, 5.8)	0.837
CRP (mg/L) ^¥^	2 (1, 4)	0 (0, 3)	0.000

Data presented as absolute values and (percentage) for female gender; mean ± standard deviation for normally distributed variables or median (25th, 75th percentile) for non-normally distributed variables. BMI—body mass index, OGTT—oral glucose tolerance test. Two groups were compared using an independent samples t-test on normally distributed data, log-transformed data (^§^), square root-transformed data (^‡^), or the Wilcoxon signed rank test (^¥^).

**Table 11:** Data for analysis in PRIMER and PERMANOVA at each taxonomic level.

	Untransformed	Square Root	Presence/Absence
Phylum			
Class			
Order	General spread	General spread	none
Family	General spread but similar to Genus		
Genus MOST DATA HIGHEST RESOLUTION	General spread—but most hopeful with just T4 visible	General spread	none
	Select > 10% contribution general spread but similar to above = any change is linked to dominant taxa shifts		
Species	Less pattern than lower taxonomic levels —potentially due to data loss		

**Table 12:** Summary of the taxonomic levels of microorganisms represented in the BioBelly gut flora data from 122 samples from 67 participants.

Row Labels	Number Categories	TOTAL Count
superkingdom	3	6,377,013
superphylum	3	1,763,398
phylum	15	6,376,988
class	25	6,376,121
order	34	6,357,174
family	71	6,089,366
genus	191	5,601,385
species	221	2,107,685
species_group	3	444
subclass	8	462,343
no_rank (total)	13	6,518,503

## Data Availability

Data are not publicly available due to participant confidentiality as defined by ethics committee. De-identified data are available on request from the corresponding author.

## Supplementary Materials

The following are available online at https://www.mdpi.com/article/10.3390/md20080500/s1, Figure S1: Flow diagram of study participants in study 1, Figure S2: Participant diagram for study 2, Figure S3: The different genera of bacteria are shown with the change in bacteria in the placebo group in blue and the change in the average active groups shown in yellow, Table S1: Comparison of Dietary Intake between three treatment groups, Table S2: Urinary F_2_-Isoprostane levels per treatment group for overweight and obese participants. Study 1, Table S3: Adherance to dietary guidelines for study population at each of the three timepoints, Table S4: blood count data from Study 1, post intervention across the three groups, including change data. Average change in before versus after for each treatment of 2 g, 4 g and placebo groups, and final levels.
