# Supplementary material for: Improved Plasma Lipids, Anti-Inflammatory Activity, and Microbiome Shifts in Overweight Participants: Two Clinical Studies on Oral Supplementation with Algal Sulfated Polysaccharide
Source: Mar Drugs. 2022 Aug 2;20(8):500. doi: 10.3390/md20080500 (PMC9410082; doi:10.3390/md20080500)
Supplement: Supplementary file 1 [file marinedrugs-20-00500-s001.zip › marinedrugs-1828867-supplementary -updated.pdf]

Article

Improved plasma lipids, anti - inflammatory activity and microbiome shifts in overweight participants: two clinical studies on oral supplementation with algal sulfated polysaccharide

Lauren Roach [1], Barbara J. Meyer [2], J. Helen Fitton and Pia Winberg

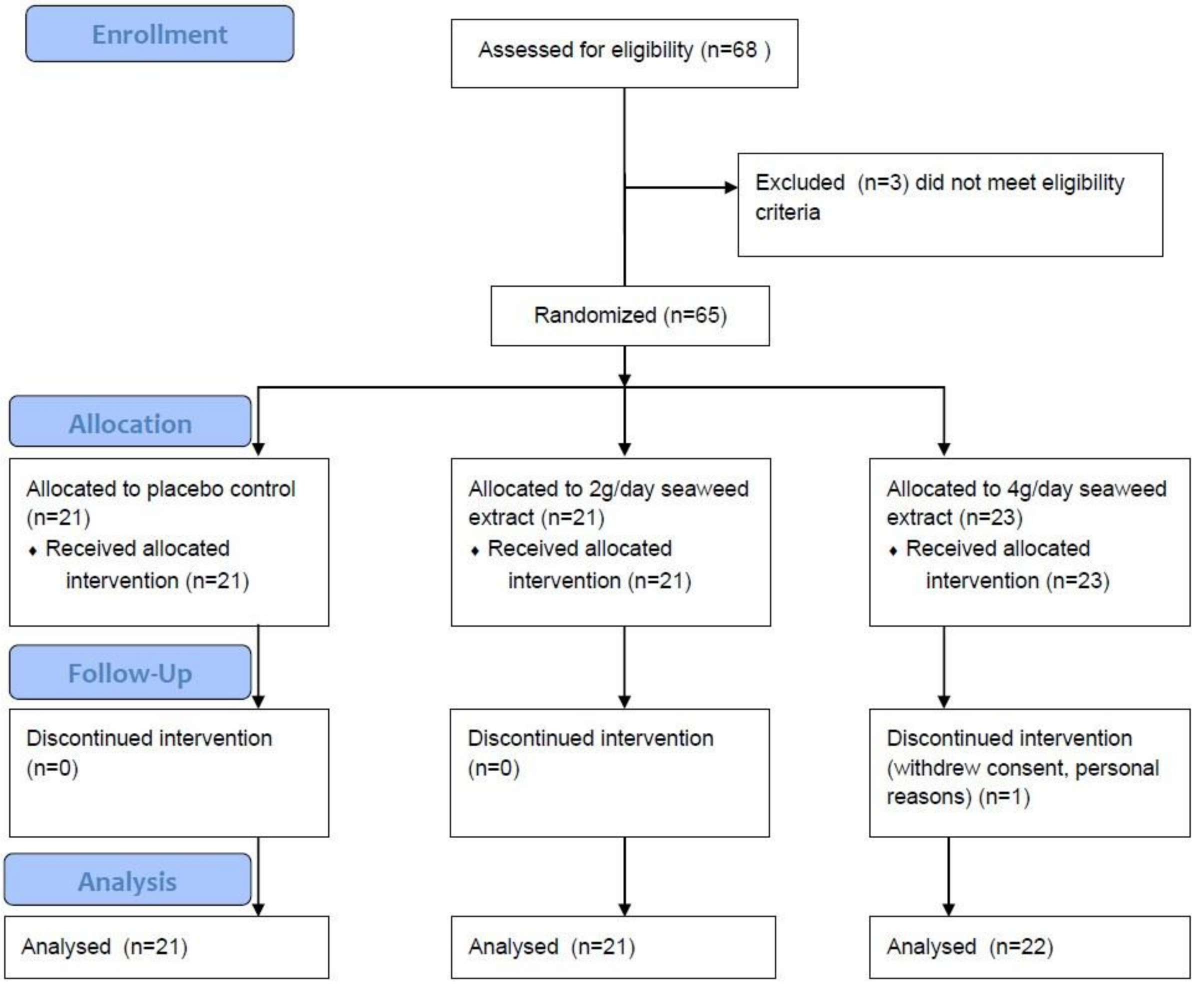

Figure S1. Flow diagram of study participants in study 1

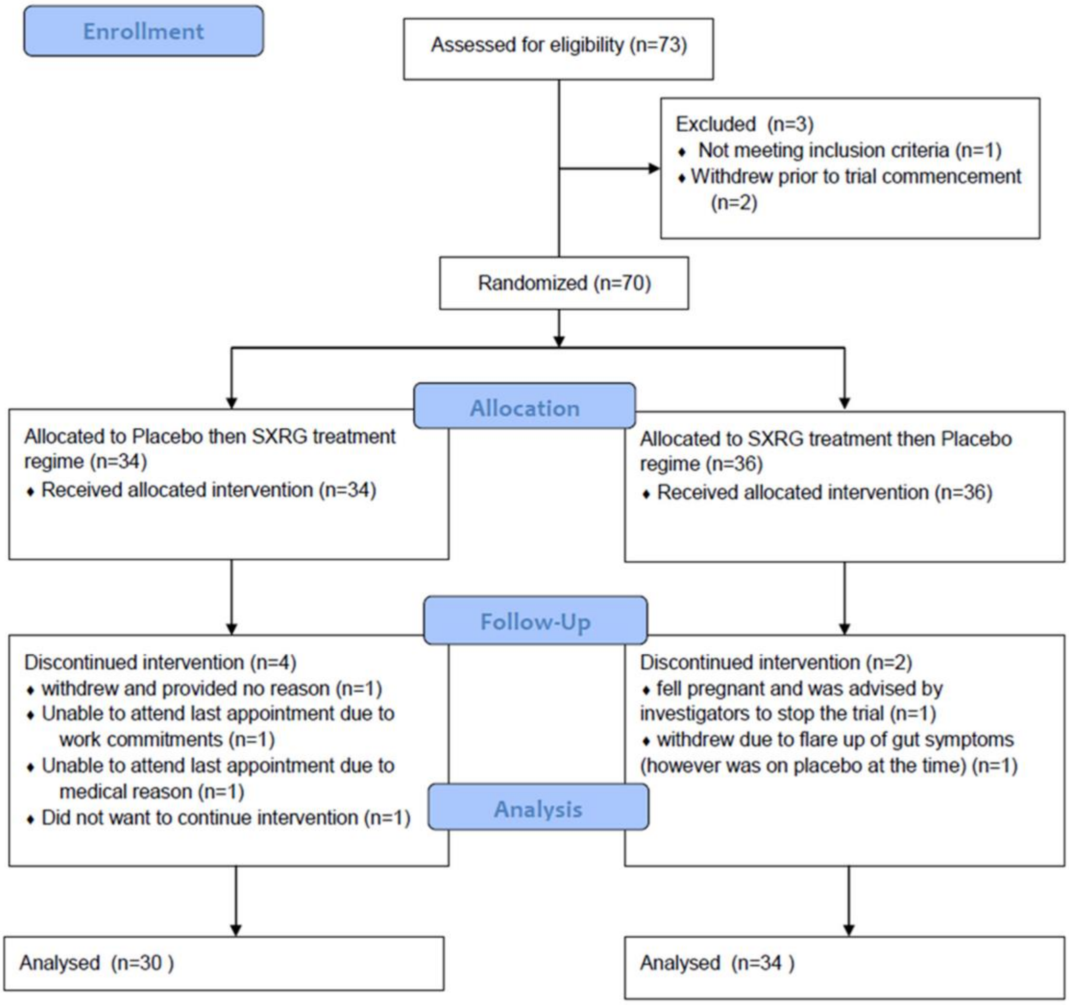

Figure S2. Participant diagram for study 2.

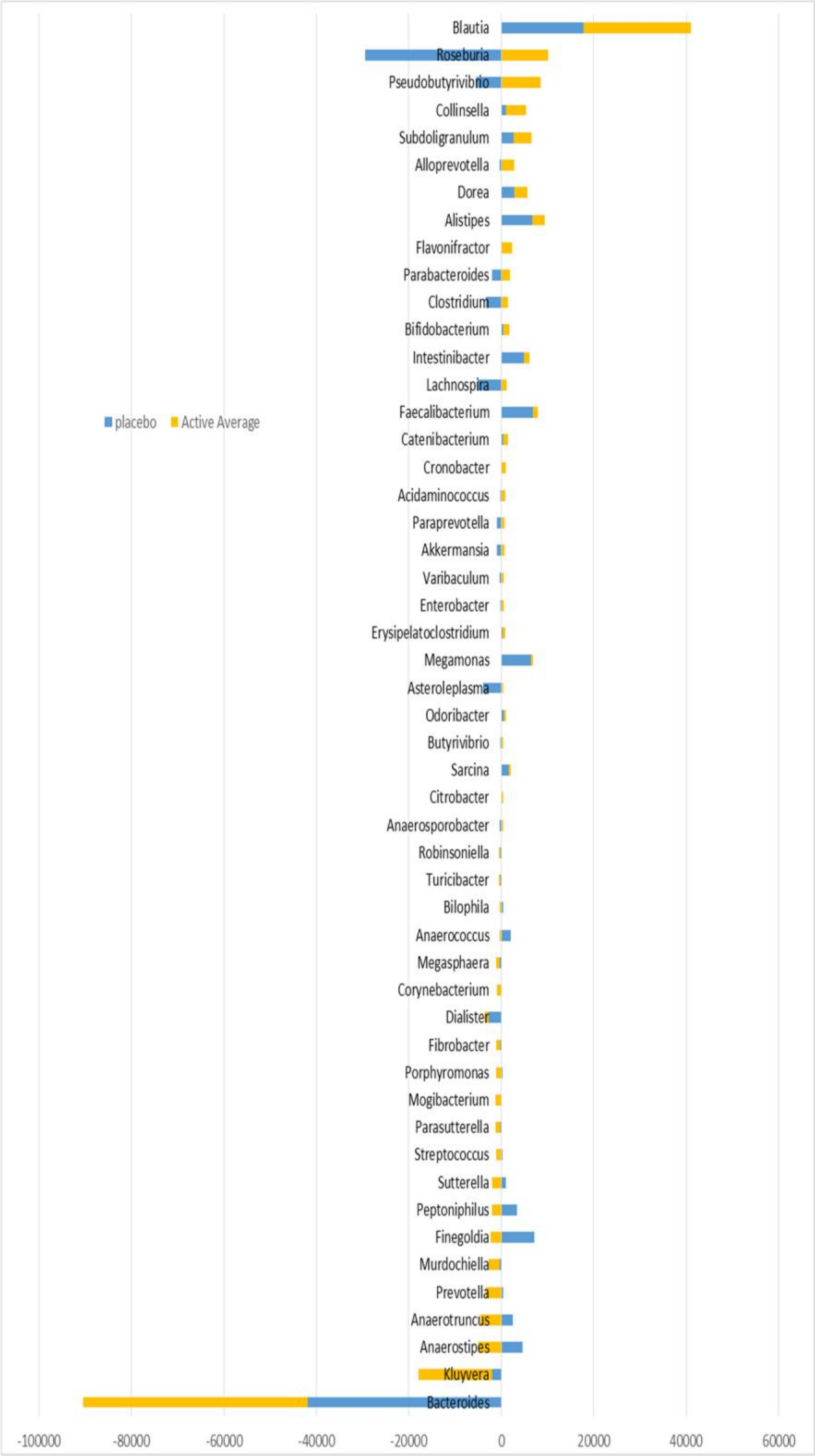

Figure S3. The different genera of bacteria are shown with the change in bacteria in the placebo group in blue and the change in the average active groups shown in yellow.

Table S1. Comparison of Dietary Intake between three treatment groups

	Placebo n=20	2g dose SXRG/day n=20	4g dose SXRG/day n=19	P Value
Total Diet Score				
Baseline	9.15 (7.92, 10.30)	9.18 (6.92, 10.36)	8.17 (6.80, 10.30)	0.456
Finish	9.13 (8.02, 10.11)	8.81 (7.04, 10.08)	8.63 (7.06, 9.80)	0.669
Change	-0.11 (-1.99, 1.83)	0.37 (-2.04, 1.62)	-0.34 (-2.06, 1.83)	0.914
Energy (kJ)				
Baseline	8500 (6864, 9474)	7946 (6251, 9028)	8303 (6575, 9999)	0.679
Finish	8575 (6656, 9495)	8284 (7181, 8762)	6996 (4942, 8508)	0.248
Change	20 (-991, 1607)	527 (-1166, 2044)	-831 (-3656, 1620)	0.162
Protein (g)				
Baseline	94 (77, 119)	91 (67, 115)	92 (85, 110)	0.757
Finish	101 (77, 123)	103 (82, 133)	99 (70, 105)	0.150
Change	7.3 (-25, 31)	19.0 (-1, 43)	2.4 (-21, 19)	0.201
Protein (%)				
Baseline	19 (17, 24)	22 (16, 24)	19 (16, 23)	0.898
Finish	22 (17, 25)	22 (19, 27)	22 (17, 25)	0.931
Change	1.6 (-3.2, 5.2)	3.4 (-3.1, 6.5)	1.9 (-1.9, 5.6)	0.837
Carbohydrate (g)				
Baseline	179 (148, 239)	189 (165, 225)	193 (148, 261)	0.993
Finish	179 (136, 235)	178 (149, 235)	143 (119, 172)	0.155
Change	-1.7 (-76, 53)	-7.3 (-53, 24)	-49 (-100, 54)	0.258
Carbohydrate (%)				
Baseline	37 (33, 39)	41 (30, 48)	38 (30, 44)	0.728
Finish	36 (33, 39)	37 (31, 39)	36 (30, 40)	0.720
Change	-0.8 (-7.9, 5.3)	-2.2 (-14, 13)	-2.3 (-11.3, 8.2)	0.819
Sugar (g)				
Baseline	89 (58, 108)	79 (49, 109)	89 (59, 108)	0.854
Finish[2]	81 (60, 96)	84 (49, 108)	55 (35, 89)	0.258
Change	-7.1 (-37, 24)	4.2 (-19, 16)	-29 (-44, 12)	0.220
Dietary Fibre (g)				
Baseline*	25 (17, 30)	24 (16, 29)	22 (15, 26)	0.399
Finish	19 (15, 26)	23 (18, 31)	22 (15, 29)	0.384
Change	-1.3 (-9.6, 0.5)	-0.1 (-5.0. 6.1)	-1.8 (-5.9, 6.1)	0.391
Total Fat (g)				
Baseline[1]	70 (58, 93)	57 (43, 84)	75 (49, 98)	0.123
Finish[1]	71 (58, 103)	75 (63, 88)	63 (52, 75)	0.170
Change	6.2 (-11, 21)	12 (-13, 35)	-8.6 (-40, 19)	0.132
Total Fat (%)				
Baseline	32 (28, 39)	28 (22, 34)	34 (28, 39)	0.150
Finish	36 (31, 41)	33 (29, 40)	34 (27, 41)	0.339
Change	1.1 (-4.4, 9.6)	6.6 (-3.5, 11)	0.3 (-8.7, 3.2)	0.302
Saturated Fat (g)				
Baseline[1]	25 (20, 32)	23 (16, 29)	24 (18, 34)	0.320
Finish[1]	25 (19, 37)	24 (21, 33)	22 (12, 30)	0.019
Change	2.1 (-6.8, 13)	2.8 (-1.2, 11)	-2.4 (-15, 4.7)	0.061
Trans Fat (g)				
Baseline[1]	1.3 (0.8, 1.6)	1.0 (0.6, 1.2)	1.0 (0.7, 1.4)	0.055
Finish[1]	1.2 (0.8, 1.8)	1.1 (0.6, 2.0)	01.0 (0.7, 1.3)	0.224
Change[1]	-0.01 (-0.4, 0.9)	0.3 (-0.2, 1.2)	-0.03 (-0.3, 0.2)	0.187
Monounsaturated Fat (g)				
Baseline[1]	25 (23, 41)	21 (15, 34)	27 (20, 37)	0.173
Finish	26 (21, 40)	30 (22, 38)	25 (20, 30)	0.379

	Placebo n=20	2g dose SXRG/day n=20	4g dose SXRG/day n=19	P Value
Change	-2.7 (-6.8, 10)	6.2 (-8.7, 17)	0.7 (-15, 7.8)	0.200
Polyunsaturated fat (g)				
Baseline[1]	11 (6.3, 14)	8.5 (5.6, 13)	11 (8.1, 17)	0.253
Finish[1]	123 (7.4, 17)	8.8 (6.5, 15)	13 (6.0, 14)	0.664
Change*	0.4 (-5.4, 5.9)	0.3 (-1.4, 3.1)	-2.3 (-5.1, 5.2)	0.773
Total Omega-6 (g)				
Baseline[1]	9.7 (5.0, 12)	6.8 (4.7, 9.6)	9.0 (6.9, 15)	0.336
Finish[1]	9.7 (5.3, 15)	7 (4.7, 13)	9.1 (5.0, 12)	0.701
Change*	-0.3 (-4.4, 5.2)	0.5 (-0.9, 2.6)	-2.0 (-3.8, 4.4)	0.785
Total Omega-3 (g)				
Baseline[1]	0.9 (0.7, 2.2)	1.5 (0.9, 2.0)	1.4 (0.8, 3.9)	0.385
Finish[1]	1.6 (0.7, 2.8)	1.2 (1.0, 1.9)	1.7 (1.0, 1.9)	0.905
Change*	0.5 (-0.3, 1.2)	-0.02 (-1.0, 0.5)	0.1 (-1.4, 0.8)	0.222
Total Long Chain Omega-3 (g)				
Baseline[1]	0.1 (0.1, 0.3)	0.2 (0.1, 0.4)	0.1 (0.1, 0.9)	0.159
Finish[1]	0.2 (0.1, 0.8)	0.1 (0.1, 0.4)	0.3 (0.1, 0.7)	0.363
Change*	0.1 (-0.1, 0.6)	-0.00 (-0.2, 0.2)	0.02 (-0.4, 0.2)	0.393
Sodium (mg)				
Baseline	1986 (1569, 2516)	1754 (1229, 2457)	2502 (1572, 3487)	0.134
Finish[2]	1941 (1655, 3556)	2295 (1436, 3220)	1917 (1459, 2828)	0.708
Change	18 (-712, 1200)	619 (-423, 1184)	-142 (-1915, 695)	0.236

Data presented as median (25th and 75th percentile)

[1] ANOVA on log transformed data

[2] ANOVA on Square root transformed data

* Kruskal Wallis test used for non-parametric data

Table S2. Urinary F2-Isoprostane levels per treatment group for overweight and obese participants. Study 1

Overweight	Placebo n=11	2g SXRG/day n=10	4g SXRG/day n=9	P Value
F_2-Isoprostane Excretion (pmol/day) baseline[1]	3938 (2472, 4538)	3709 (2742, 4954)	4023 (2622, 5365)	0.893
F_2-Isoprostane Excretion (pmol/day) post[1]	4001 (2513, 6399)	3500 (2738, 3691)	4482 (2505, 5955)	0.474
F_2-Isoprostane Excretion (pmol/day) (post-baseline)*	980 (121, 1815)	-925 (-1268, 1126)	297 (-1500, 1119)	0.233
Obese	Placebo n=9	2g SXRG/day n=9	4g SXRG/day n=12	P Value
F_2-Isoprostane Excretion (pmol/day) baseline[1]	4641 (3714, 7038)	5579 (2193, 7064)	5301 (4811, 7591)	0.551
F_2-Isoprostane Excretion (pmol/day) post[1]	4977 (2995, 7306)	4026 (2477, 7640)	6952 (4086, 7679)	0.312
F_2-Isoprostane Excretion (pmol/day) (post-baseline)*	319 (-2483, 1742)	-78 (-1894, 646)	-269 (-1068, 1992)	0.811

Data presented as median (25th and 75th percentile)

[1] ANOVA on log transformed data

* Kruskal Wallis test used for non-parametric data

Table S3. Adherance to dietary guidelines for study population at each of the three timepoints

Nutrient	EAR Male	% Male T1 (n=30)	% Male T2 (n=29)	% Male T3 (n=30)	EAR Female	% Female T1 (n=31)	% Female T2 (n=33)	% Female T3 (n=31)
Protein	52g	83	86	80	37g	97	94	97
Thiamin	1mg	87	79	77	0.9mg	74	79	71
Riboflavin	1.1mg	93	79	80	0.9mg	97	97	97
Niacin.	12mg	100	100	97	11mg	100	100	100
Vitamin C	30mg	100	97	83	30mg	100	97	90
Vitamin B6	1.1mg (19-50 yo)<br>1.4mg (> 50 yo)	92<br>33	83<br>53	92<br>67	1.1mg (19-50 yo)<br>1.3mg (51 + yo)	87<br>56	75<br>47	69<br>73
Vitamin B12	2µg	97	93	90	2µg	94	94	97
Folate	320µg	97	90	77	320µg	90	91	90
Vitamin A	625µg	73	52	53	500µg	90	82	94
Magnesium	350mg	40	52	63	265mg	90	82	84
Calcium	840mg (19-70yo)<br>1100mg (>70 yo)	46<br>33	35<br>33	25<br>17	840mg (19-50yo)<br>1100mg (>50 yo)	47<br>19	44<br>24	44<br>20
Phosphorus	580mg	100	100	97	580mg	100	97	97
Iron	6mg	100	93	90	8mg (19-50 yo)<br>5mg (>50 yo)	73<br>100	81<br>100	75<br>100
Zinc	12mg	33	45	43	6.5mg	90	97	94
Selenium	60µg	90	86	94	50µg	90	91	84
Iodine	100µg	80	86	70	100µg	84	88	90

Data presented as percentage of participants meeting the estimated average Requirement (EAR) at each study timepoint.
EAR, estimated average requirement. T1= baseline, T2= 6 weeks, T3= 12 weeks.

Table S4. blood count data from Study 1, post intervention across the three groups, including change data. Average change in before versus after for each treatment of 2g, 4g and placebo groups, and final levels

	Placebo	Delta placebo	2g	Delta 2g	4g	Delta 4g
Final Haemoglobin (g/L)	138.4	2.7	141.7	1.8	139.8	2.5
Final RCC (10^12/L)	4.7	0.1	4.7	0.1	4.7	0.1
Final Haematocrit	0.4	0.0	0.4	0.0	0.4	0.0
Final MCV (fL)	88.6	-0.3	90.9	-0.8	89.8	-0.5
Final MCH (pg)	29.3	-0.1	30.4	0.1	29.6	0.0
Final MCHC (g/L)	330.3	-0.1	335.0	3.1	330.1	1.9
Final RDW	13.7	0.0	13.6	-0.1	13.5	-0.5
Final WCC (10^9/L)	6.0	-0.3	6.4	-0.1	6.6	0.6
Final Neutrophils (10^9/L)	3.2	-0.2	3.7	-0.2	3.1	0.1
Final Lymphocytes (10^9/L)	2.1	-0.1	2.0	0.1	2.8	0.5
Final Monocytes (10^9/L)	0.4	0.0	0.5	0.0	0.5	0.0
Final Eosinophils (10^9/L)	0.2	-0.2	0.2	-0.2	0.2	-0.2
Final Basophil (10^9/L)	0.1	0.0	0.1	0.0	0.1	0.0
Final Platelets (10^9/L)	245.7	9.9	234.1	1.5	238.9	7.3

CONSORT 2010 checklist of information to include when reporting a randomised trial*

Section/Topic	Item No	Checklist item	Reported on page No
Title and abstract			
	1a	Identification as a randomised trial in the title	112
	1b	Structured summary of trial design, methods, results, and conclusions (for specific guidance see CONSORT for abstracts)	112
Introduction			
Background and objectives	2a	Scientific background and explanation of rationale	113-114
	2b	Specific objectives or hypotheses	113-114
Methods			
Trial design	3a	Description of trial design (such as parallel, factorial) including allocation ratio	114
	3b	Important changes to methods after trial commencement (such as eligibility criteria), with reasons	NA
Participants	4a	Eligibility criteria for participants	114
	4b	Settings and locations where the data were collected	115
Interventions	5	The interventions for each group with sufficient details to allow replication, including how and when they were actually administered	116
Outcomes	6a	Completely defined pre-specified primary and secondary outcome measures, including how and when they were assessed	116
	6b	Any changes to trial outcomes after the trial commenced, with reasons	NA
Sample size	7a	How sample size was determined	114
	7b	When applicable, explanation of any interim analyses and stopping guidelines	NA
Randomisation:			
	8a	Method used to generate the random allocation sequence	114

Sequence generation	8b	Type of randomisation; details of any restriction (such as blocking and block size)	114
Allocation concealment mechanism	9	Mechanism used to implement the random allocation sequence (such as sequentially numbered containers), describing any steps taken to conceal the sequence until interventions were assigned	114
Implementation	10	Who generated the random allocation sequence, who enrolled participants, and who assigned participants to interventions	114
Blinding	11a	If done, who was blinded after assignment to interventions (for example, participants, care providers, those assessing outcomes) and how	114, 117
	11b	If relevant, description of the similarity of interventions	NA
Statistical methods	12a	Statistical methods used to compare groups for primary and secondary outcomes	117
	12b	Methods for additional analyses, such as subgroup analyses and adjusted analyses	117
Results			
Participant flow (a diagram is strongly recommended)	13a	For each group, the numbers of participants who were randomly assigned, received intended treatment, and were analysed for the primary outcome	118
	13b	For each group, losses and exclusions after randomisation, together with reasons	118
Recruitment	14a	Dates defining the periods of recruitment and follow-up	115
	14b	Why the trial ended or was stopped	NA
Baseline data	15	A table showing baseline demographic and clinical characteristics for each group	119
Numbers analysed	16	For each group, number of participants (denominator) included in each analysis and whether the analysis was by original assigned groups	119-121
Outcomes and estimation	17a	For each primary and secondary outcome, results for each group, and the estimated effect size and its precision (such as 95% confidence interval)	119-124
	17b	For binary outcomes, presentation of both absolute and relative effect sizes is recommended	NA
Ancillary analyses	18	Results of any other analyses performed, including subgroup analyses and adjusted analyses, distinguishing pre-specified from exploratory	NA
Harms	19	All important harms or unintended effects in each group (for specific guidance see CONSORT for harms)	NA
Discussion			

Limitations	20	Trial limitations, addressing sources of potential bias, imprecision, and, if relevant, multiplicity of analyses	129
Generalisability	21	Generalisability (external validity, applicability) of the trial findings	128
Interpretation	22	Interpretation consistent with results, balancing benefits and harms, and considering other relevant evidence	125-130
Other information			
Registration	23	Registration number and name of trial registry	114
Protocol	24	Where the full trial protocol can be accessed, if available	NA
Funding	25	Sources of funding and other support (such as supply of drugs), role of funders	174

*We strongly recommend reading this statement in conjunction with the CONSORT 2010 Explanation and Elaboration for important clarifications on all the items. If relevant, we also recommend reading CONSORT extensions for cluster randomised trials, non-inferiority and equivalence trials, non-pharmacological treatments, herbal interventions, and pragmatic trials. Additional extensions are forthcoming: for those and for up to date references relevant to this checklist, see www.consort-statement.org.
